# High-efficiency and flexible organic solar modules with promising applications in solar-extended unmanned aerial vehicles

**DOI:** 10.1093/nsr/nwaf519

**Published:** 2025-11-19

**Authors:** Chenyang Tian, Hao Zhang, Ziqi Zhang, Caixuan Wang, Dan Deng, Dingding Qiu, Jing Tao, Kamran Amin, Kaiwu Peng, Jia Li, Tong Wang, Yuhan Wang, Jianqi Zhang, Zhixiang Wei

**Affiliations:** CAS Key Laboratory of Nanosystem and Hierarchical Fabrication, National Center for Nanoscience and Technology, Chinese Academy of Sciences, Beijing 100190, China; School of Nanoscience and Technology, University of Chinese Academy of Sciences, Beijing 100049, China; CAS Key Laboratory of Nanosystem and Hierarchical Fabrication, National Center for Nanoscience and Technology, Chinese Academy of Sciences, Beijing 100190, China; Ningbo Institute of Materials Technology and Engineering, Chinese Academy of Sciences, Ningbo 315201, China; CAS Key Laboratory of Nanosystem and Hierarchical Fabrication, National Center for Nanoscience and Technology, Chinese Academy of Sciences, Beijing 100190, China; School of Nanoscience and Technology, University of Chinese Academy of Sciences, Beijing 100049, China; CAS Key Laboratory of Nanosystem and Hierarchical Fabrication, National Center for Nanoscience and Technology, Chinese Academy of Sciences, Beijing 100190, China; CAS Key Laboratory of Nanosystem and Hierarchical Fabrication, National Center for Nanoscience and Technology, Chinese Academy of Sciences, Beijing 100190, China; CAS Key Laboratory of Nanosystem and Hierarchical Fabrication, National Center for Nanoscience and Technology, Chinese Academy of Sciences, Beijing 100190, China; School of Nanoscience and Technology, University of Chinese Academy of Sciences, Beijing 100049, China; CAS Key Laboratory of Nanosystem and Hierarchical Fabrication, National Center for Nanoscience and Technology, Chinese Academy of Sciences, Beijing 100190, China; CAS Key Laboratory of Nanosystem and Hierarchical Fabrication, National Center for Nanoscience and Technology, Chinese Academy of Sciences, Beijing 100190, China; Ningbo Institute of Materials Technology and Engineering, Chinese Academy of Sciences, Ningbo 315201, China; CAS Key Laboratory of Nanosystem and Hierarchical Fabrication, National Center for Nanoscience and Technology, Chinese Academy of Sciences, Beijing 100190, China; CAS Key Laboratory of Nanosystem and Hierarchical Fabrication, National Center for Nanoscience and Technology, Chinese Academy of Sciences, Beijing 100190, China; School of Nanoscience and Technology, University of Chinese Academy of Sciences, Beijing 100049, China; CAS Key Laboratory of Nanosystem and Hierarchical Fabrication, National Center for Nanoscience and Technology, Chinese Academy of Sciences, Beijing 100190, China; CAS Key Laboratory of Nanosystem and Hierarchical Fabrication, National Center for Nanoscience and Technology, Chinese Academy of Sciences, Beijing 100190, China; School of Nanoscience and Technology, University of Chinese Academy of Sciences, Beijing 100049, China

**Keywords:** organic solar cells, power conversion efficiency, flexible solar cells, slot-die coating, solar power management

## Abstract

Organic solar cells (OSCs) offer unique advantages like flexibility and lightweight design, making them suitable for solar-extended unmanned aerial vehicles (SUAVs). However, conventional transparent electrodes limit their performance due to high sheet resistance. To address this, a flexible, transparent electrode with ultra-low sheet resistance (<1 Ω/□) and 90% transmission was developed. Utilizing non-halogenated solvent processing and slot-die coating, a 1 cm^2^ single cell achieved 17.12% (certified 16.88%) power conversion efficiency (PCE), while a 42 cm^2^ module achieved 15.60%. Stability tests showed unencapsulated devices retained 90% efficiency after 1080 h (ISOS-D-1) and 97% after 1000 bending cycles. SUAVs equipped with the flexible OSC modules, combined with a lithium battery and power management system, demonstrated a flight time extension of 24.2%. Outdoor testing confirmed reliable sensor performance and data transmission. This study validates flexible OSCs for SUAV applications, advancing renewable energy solutions in lightweight mobile systems.

## Teaser

Flexible organic solar cells featuring high-transparency, low-resistance electrodes have achieved record-breaking efficiency and durability, demonstrating significant potential for powering lightweight, solar-extended unmanned aerial vehicles.

## INTRODUCTION

Solar-extended unmanned aerial vehicles (SUAVs), equipped with long endurance and onboard environmental sensors, are ideal for applications such as surveillance, remote sensing and infrastructure inspection [1,2]. Additionally, SUAVs provide unique advantages for atmospheric monitoring by delivering high-resolution, real-time data on temperature, humidity and wind across various altitudes in the planetary boundary layer, filling critical observational gaps in areas inaccessible to traditional systems, and enhancing the accuracy of weather predictions [3,4]. Unlike traditional crystalline silicon and Cu(In,Ga)Se_2_ (CIGS) solar cells [5,6], flexible organic solar cells (OSCs) are particularly advantageous for providing energy to self-powered drones, sounding balloons and near-space aircraft due to their lightweight and flexible nature [7,8]. In addition, OSCs offer advantages such as flexibility, compatibility with roll-to-roll production processes, and the ability to conform to various curved surfaces.

The benefits enable OSCs to be integrated into energy storage systems for continuous power supply in off-grid scenarios [9]. For instance, researchers have developed milliwatt-level flexible OSC modules integrated with flexible energy storage devices to power low-energy systems like robotic insects and wearable sensors [10–12]. However, the low efficiency of these OSC modules limits their applications to low-power scenarios, making them incapable of driving high-power loads. Encouragingly, the efficiency of small-area OSCs has recently exceeded the 20% milestone [13–15], providing a solid foundation for scaling up to high-efficiency large-area modules. The development progress of flexible large-area single-cell OSCs in recent years is summarized in Fig. S1. However, the cutting-edge results demonstrate that the scale-up of flexible OSCs is accompanied by a significant decline in performance, which can be attributed to the electrical loss associated with the large sheet resistance of the flexible transparent conductive electrode (TCE).

An ideal TCE should exhibit high transmittance, low resistance and long-term stability in outdoor environments. Materials such as transparent conductive oxides, metal nanowires and conductive polymers have been used to meet such demand [16–18]. Indium tin oxide (ITO) and silver nanowires offer a good balance between conductivity and light transmission, but ITO is brittle and has poor flexibility. Silver nanowires are more flexible but have a morphology that carries the potential of piercing the active layer, and dramatically reduces the shunt resistance of the device [19]. In contrast, transparent metal grid electrodes can achieve lower sheet resistance at the same transmittance and maintain excellent bending stability [20]. However, metal grids often struggle with difficulties brought by excessive roughness and parasitic lateral current flow during subsequent film depositions. Additionally, a conductive smoothing layer is usually required to improve charge transport and collection in large areas between grids [21]. The conductive polymer PH1000 is commonly used as a metal grid smoothing layer due to its high optical transmittance and compatibility with solution processing. However, its hygroscopic nature and acidity limit the long-term stability of the device, which urgently needs improvement [22–24].

Here, we replaced traditional PH1000 with a low-temperature sputtered ITO, eliminating the need for thermal annealing. This substitution maintains high transmittance and low sheet resistance, while reducing metal oxide brittleness. The device retained 97% of initial efficiency after 1000 bends and demonstrated enhanced environmental stability, overcoming the susceptibility of PH1000 to moisture. LITO-based devices maintained 89.6% efficiency after 1080 h under the ISOS-D-1 standard protocol, significantly improving durability. Integrating silver–copper (AgCu) composites with LITO also enhanced processing uniformity, achieving power conversion efficiencies (PCEs) of 17.12% (certificated 16.88%) for 1 cm^2^ cells and 15.60% for 42 cm^2^ modules. Finally, flexible OSC modules combined with lithium batteries formed a stable solar-storage hybrid system in an SUAV, performing reliably during charge–discharge cycles and successfully transmitting flight data, confirming the feasibility of OSC-powered drones for environmental monitoring and remote sensing, thus broadening flexible OSC applications in off-grid systems.

## RESULTS AND DISCUSSION

### Large-area slot-die coating uniformity and performance of flexible OSC modules

**Figure 1.**
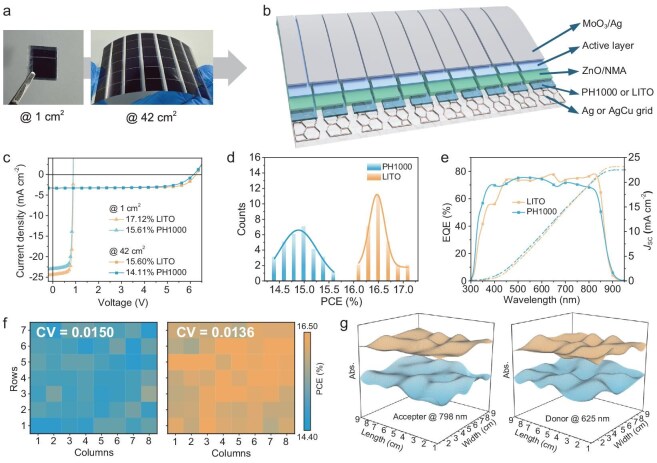
Flexible OSC structures and the characteristics of device repeatability and uniformity. (a) Images of the single cell and the module. (b) Schematic diagram of the OSC module structure and series connection. (c) *J–V* curves of optimized devices based on PH1000 and LITO modified, respectively. (d) Statistical PCE data of more than 25 individual 1 cm^2^ single cells. The bar graph shows the efficiency statistics, and the solid line is the Gaussian fit of the statistical data. (e) EQE curves of devices based on PH1000 and LITO modified, respectively. (f) Heatmap of the PCE of 7 × 8 array 1 cm^2^ individual devices from one substrate. The coefficient of variation (CV) is marked in the upper left. The left chart is PH1000-modified results, while the right-side corresponds to LITO-modified ones. (g) Film uniformity based on the mapping of UV–vis absorption spectra. The absorption peak intensities of the donor at 625 nm and the acceptor at 798 nm were marked as indicators. The orange-colored graph represents data for LITO, while the blue-colored graph corresponds to PH1000.

Figure 1a shows actual photographs of flexible OSC devices. On the left is a single-cell device with an area of 1 cm^2^, while the right image depicts an enlarged flexible OSC module with an area of 42 cm^2^, consisting of seven sub-cells. Each sub-cell measures 1 cm in width and 6 cm in length. Figure 1b illustrates the device architecture of the OSC module. The flexible substrate is made of polyethylene terephthalate (PET) with a thickness of 125 µm. On top of the PET substrate, a honeycomb-patterned metal grid is fabricated using photolithography and nanoimprinting techniques [25]. The grid has a side length of 100 µm (Fig. S2). In this study, two types of metal fillers were selected: pure silver and an AgCu composition, each with different conductivities and transmittances, which will be discussed in detail later.

On top of the metal grid substrate, successive layers were printed using a slot-die coating process: an electrode modification layer (the LITO layer was deposited via magnetron sputtering), followed by an electron transport layer, and an active layer [26,27]. The molecular structures of the solution-processed materials are provided in Fig. S3, with detailed processing parameters described in the Materials and Methods section. The final layers, comprising the hole transport layer (MoO_3_) and the Ag electrode, were deposited via thermal evaporation, and a mask was used to pattern the module, forming seven sub-cells connected in series. A schematic diagram illustrating the module dimensions and electrical interconnection configuration is provided in Fig. S4. The geometry fill factor (GFF) of the flexible module is 78%. Under the AM 1.5G illumination, the photovoltaic *J–V* characteristics of both the 1 cm^2^ single device and the 42 cm^2^ module are measured.

The molecular system used in this study is the previously reported PM6:TrimerC8C10 [27]. To improve the short-circuit current density (*J*_SC_), a third component N3, was added, and the ratio of N3 (Table S1) and D:A (Table S2) was optimized. The results showed that the optimal conditions were achieved with an N3 content of 10%, a D:A ratio of 1.5, and a total solution concentration of 21.2 mg/mL. By using the solid additive 1,4-diiodobenzene (DIB) instead of the traditional 1-chloronaphthalene (CN), reduced carrier recombination and more efficient charge transport were achieved [28], resulting in an increase in the device PCE from 15.1% to 15.6% (Table S3). The above data were presented based on the silver/PH1000 substrate.

When the TCEs were optimized to the AgCu composite electrode modified with LITO, the device performance was significantly improved. The best device performance is shown in Fig. 1c, where the LITO-modified 1 cm^2^ device achieved a PCE of 17.12%. The detailed device parameters include an open-circuit voltage (*V*_OC_) of 0.895 V, a *J*_SC_ of 24.37 mA/cm^2^, and a fill factor (FF) of 78.42%. The corresponding current density integrated from the external quantum efficiency (EQE) data is 23.19 mA/cm^2^, within a 5% deviation from the measured current density. The device efficiency was certified (certification report shown in Fig. S5), with the device parameters as follows: *V*_OC_ = 0.886 V, *J*_SC_ = 24.86 mA/cm^2^, FF = 76.65% and PCE = 16.88%. The 42 cm^2^ module attained an efficiency of 15.60% (*V*_OC_ = 6.247 V, *J*_SC_ = 3.28 mA/cm^2^, FF = 76.18%). In comparison, devices with the conventional PH1000 electrode modification layer achieved efficiencies of 15.61% and 14.11%, respectively. The PCE values for both the single-cell device and the module represent the highest reported efficiencies for flexible OSCs processed using non-halogenated solvents (Fig. S1). Statistical data on device efficiency are listed in Tables S4, S5 and Fig. 1d. The LITO modification led to an increase in the short-circuit current by 1.34 mA/cm^2^, as further confirmed by the EQE data in Fig. 1e. The optimized device exhibited significantly enhanced PCE, particularly in the 600–820 nm wavelength range.

In addition to the improvement in the optimal device performance, the repeatability and uniformity of PCE with LITO modification substrate were also enhanced. As shown in Fig. 1d, the PCE distribution of the 1 cm^2^ devices indicates that the average efficiency increased from 14.9% to nearly 16.5%, while the standard deviation decreased from 0.33 to 0.27. Statistical analysis was conducted on a 7 × 8 array of 56 individual devices cutting from a single large-area substrate (8 × 9 cm), each with an area of 1 cm^2^, as shown in Fig. 1f and Fig. S6. We used the coefficient of variation (CV) to examine the fluctuation within each dataset [29]. Devices utilizing LITO as the electrode modification material exhibited better uniformity, indicating that scaling the device area to the full-size design could result in higher module efficiency. Correspondingly, compared to the average efficiency of the 42 cm^2^ module in Table S4, the AgCu/LITO substrate achieved an improvement of nearly 10.6%.

Figure 1g further confirms this through variations in the film absorption spectrum. Using a fiber spectrometer, we conducted rapid point-by-point scanning of the absorption spectrum across the entire flexible substrate coated with the active layer film. The absorption peaks at 625 nm (donor) and 798 nm (acceptor) in the blend film were chosen to characterize the variation in film absorption. Due to the evaporation shadowing effect of the mask, the array total area is smaller than the total active layer area on the substrate, so the film absorption spectrum scanning covered a region of 8 × 9 cm. Here, we are not concerned with absolute absorption values but rather with the degree of variation. The CV values for the two absorption peaks in the LITO-modified films were 0.0107 and 0.0129, respectively. In contrast, the CV values for the PH1000-based films were 0.0217 and 0.0216. The results demonstrate that the active layer shows a notable homogeneity enhancement when an LITO-modified substrate is employed. This evidence substantiates the assertion that substrates modified with LITO offer a substantial advantage in the processing of large-area OSC modules.

### Analysis of the characteristics of TCEs

The efficiency variations in flexible photovoltaic devices are closely related to the performance of the TCE. As mentioned above, two types of metal fillers, silver grid and AgCu composite grid, were used for the metal grid electrodes in the experiment. The silver grid substrate had a surface height difference of nearly 500 nm between the conductive grooves and the rest of the substrate due to the limited metal fill height [30]. A conductive polymer, PH1000, was used to smooth the surface to cover the substrate. Although using a AgCu composite filler optimized the height difference to around 50 nm, the device would be short-circuited without an electrode modification layer [31]. Given the hygroscopic and acidic nature of PH1000, the oxidation of the AgCu composite electrode is likely to be accelerated by PH1000, resulting in a rapid increase in sheet resistance. Consequently, the metal oxide LITO was selected as a replacement for PH1000.

The In/Sn ratio in ITO thin films is a key determinant of their optical and electrical properties. While increasing the Sn concentration enhances conductivity and reduces sheet resistance, excessive Sn content can diminish transmittance due to absorption or scattering defects [32,33]. Commercial ITO electrodes typically optimize the In/Sn ratio to 9, achieving a balance between conductivity and transmittance [32,33]. In contrast, LITO films deposited on PET substrates adopt an In/Sn ratio of 13.5, specifically designed to enhance long-wavelength transmittance while maintaining excellent overall performance (Figs S7–S9). X-ray photoelectron spectroscopy (XPS) spectra analysis reveals significant differences in the relative intensities of In 3d and Sn 3d characteristic peaks (Fig. S10). When combined with AgCu electrodes, LITO achieves superior performance without significantly increasing sheet resistance, resulting in an advanced composite electrode. We evaluated LITO layers of varying thicknesses. Although a thickness of 20 nm achieved the highest transmittance, it caused a 58% increase in sheet resistance (*R*_sh_) (Fig. S8 and Table S6). The crystallinity of ITO sputtered on glass, PET/ITO and PET/LITO was analyzed using grazing-incidence wide-angle X-ray diffraction (GIWAXS). The 2D GIWAXS images are summarized in Fig. S11. It was revealed that there were three distinct diffraction rings at q-vectors of 1.53, 2.16 and 2.45 Å^−1^, corresponding to the diffraction peaks of (211), (222) and (400) crystal planes of crystalline ITO [34]. The 2D diffraction data were integrated to obtain a plot of diffraction angle versus diffraction signal intensity (Fig. S11). There were no distinct diffraction peaks corresponding to the crystalline ITO observed for the two types of ITO on the PET substrate, indicating that the ITO deposited on PET is amorphous. According to literature reports, the three peaks at 2*θ* angles of 17.5°, 22.6° and 26.0° can be attributed to the orientation peaks of PET [35].

**Figure 2.**
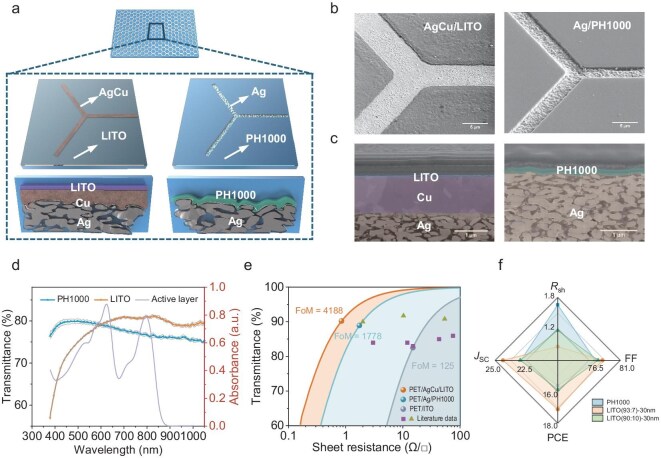
Morphology, uniformity and optical-electrical characterization of TCEs. (a) Schematic diagrams of TCEs based on PH1000 and LITO modified, respectively. (b) 52° tilt SEM images of TCEs. The scale bars are 5 μm. (c) Cross-section SEM images of metal grids. The scale bars are 1 μm. (d) Transmission spectra of nine regions of TCEs and absorption spectra of the active layer film. The light gray line is the error band. (e) The *FoM* comparison of three types of TCEs. The green triangles represent transmittance data excluding the substrate. The purple squares represent transmittance data including the substrate. (f) Device performance comparison based on the three TCEs.

Figure 2a and b provides schematic diagrams and 52° tilt scanning electron microscope (SEM) images of the transparent conductive substrates AgCu/LITO and Ag/PH1000, respectively. Despite using PH1000 to fill the depressions in the metal grooves, the conductive grid still sits lower than the substrate surface, contrary to expectations. In contrast, the surface of the AgCu composite electrode becomes smoother after LITO modification. Atomic force microscopy (AFM) was employed to characterize the surface morphology of the composite electrodes, enabling evaluation of surface uniformity and topographical variation over a larger area. As shown in Fig. S12, the height difference between the electrode grid and the underlying substrate was measured. For the LITO-modified electrode, the height difference was approximately 35 nm, whereas that of the PH1000-modified electrode reached a maximum exceeding 200 nm. Further comparison of surface roughness revealed that the LITO-modified electrode exhibits significantly improved surface uniformity and enhanced grid filling capability. Using focused ion beam (FIB) technology, we cut through the transparent electrode and captured cross-sectional images with SEM, which are shown in Fig. 2c. Detailed information is provided in the Materials and Methods section. The silver paste layer, copper deposition layer and modification layers (LITO or PH1000) are color-coded. Above the modification layer is a platinum protective layer deposited prior to ion etching. The LITO layer exhibited more uniform deposition, with a measured thickness of approximately 30 nm from the cross-sectional SEM. However, the PH1000 layer showed significant height fluctuations. A magnified image of the metal grid area (Fig. S13) reveals a rough network of PH1000 fibers and black voids left by solvent evaporation. These defects affected the subsequent ZnO/NMA electron transport layer deposition (Fig. S14), further impacting film uniformity.

We measured the *R*_sh_ of nine regions on substrates prepared under two different conditions (Fig. S15). The *R*_sh_ variation for PH1000 was significantly larger, ranging from 1.17 to 1.93 Ω/□, while the *R*_sh_ for LITO substrates not only decreased by nearly 50%, ranging from 0.83 to 0.90 Ω/□, but also showed more uniformity across different regions. Meanwhile, the coefficients of variation for the *R*_sh_ are 0.1212 and 0.0290, respectively, demonstrating the difference in uniformity between the two modifications. The transmittance spectra from the same nine regions were also collected and plotted; see Fig. 2d. The colored datapoints represent the average values of the spectra, with the gray shaded areas indicating the error range, and the solid red line representing the absorption spectrum of the blend film. The transmittance spectra were not corrected for PET substrate absorption (Fig. S7). The PH1000-modified transparent substrate achieved a maximum transmittance of 80% at 495 nm, while the LITO-based substrate had a peak transmittance of 81% at 796 nm. Although PH1000 exhibited higher transmittance below 590 nm (corresponding to the higher photo response of the PH1000 device below 600 nm, as shown in Fig. 1e), the LITO substrate had higher transmittance in the absorption range of both the donor and acceptor. The different modification layers deposited individually on the PET substrate also exhibited similar patterns to those of the composite electrode (Fig. S9 and Table S7). We compared four different modification layers and found that the LITO (93:7) (the proportion here indicates a composition of 93% indium and 7% tin) with a thickness of 30 nm provided a balanced *J*_SC_ and FF, leading to the optimal device performance (Table S8). Compared to commercial PET/ITO (*R*_sh_ = 15 Ω/□), while the transmittance of the modified metal grid electrodes was somewhat lower (Fig. S7), their conductivity was significantly improved. According to the relationship equation between transmittance and *R*_sh_ in thin metallic films [36], where *σ*_Op_(*λ*) is the optical conductivity and *σ*_DC_ is the conductivity of the film, here we used the transmittance data at 625 nm because of the absorption peak of PM6. A figure of merit (*FoM*), defined as *σ*_DC_/*σ*_Op_, was introduced to evaluate the balance between transmittance and conductivity in the TCE (Note S1). A comparison of the three substrates (Fig. 2e) shows that the LITO-modified substrate slightly improved transmittance while significantly enhancing conductivity. Compared to the PET/ITO substrate, the *FoM* value increased by approximately 34 times, reaching 4188. This value offers a significant advantage over other types of TCEs [17–19,37–45]. A comparison of performance metrics with state-of-the-art TCEs is provided in Table S9. Based on Ag/PH1000 substrates and AgCu transparent electrode substrates modified under various LITO deposition conditions, we fabricated flexible devices to compare their performance differences; see Fig. 2f (detailed device parameters are listed in Tables S5 and S7). Benefiting from the enhanced transmittance and conductivity provided by the LITO modification layer, significant improvements in *J*_SC_ and FF were observed. The optimized condition of 30 nm LITO (93:7) condition yielded the best device performance among different modification layers.

### Bending and environmental stabilities of the flexible OSCs

**Figure 3.**
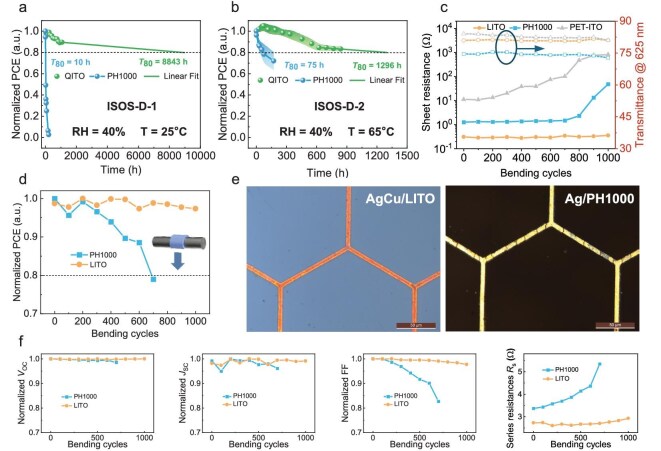
Comparison of device and TCE stabilities based on different electrode modification. (a) The environmental stability test based on ISOS-D-1 protocol; the average test temperature is 25°C and the relative humidity is 40%. (b) The thermal stability test based on ISOS-D-2 protocol; the heating temperature is 65°C. (c) The changes in sheet resistance and transmittance of TCEs during the bending test (solid line and symbols represent sheet resistance data, dashed line and hollow symbols represent the transmittance of the TCEs at 625 nm). The bending radius is 5 mm. (d) Bending test with a radius of 5 mm. The inset schematic diagram shows that the device is downward-curved close to the surface of the metal cylinder. (e) The images of the TCEs under an optical microscope after 1000 bending cycles. The left side is AgCu/LITO, and the right side is Ag/PH1000. The scale bar is 50 μm. (f) The variation of device parameters (*V*_OC_, *J*_SC_, FF and *R*_s_) during bending test.

The stability of unencapsulated 1 cm^2^ single-cell devices was evaluated under ambient conditions and at 65°C, following the test protocols defined by the ISOS-D-1 and ISOS-D-2 protocols [46,47]. The relative humidity (RH) in the testing laboratory was maintained at 40%. Five independent devices were selected for each test condition, and the stability test results were plotted; see Fig. 3a and b. Each datapoint represents the average value of the five devices, and the shaded areas indicate the error margins.

For stability at room temperature, devices modified with LITO showed significantly better performance than those with PH1000. After 1080 h of testing, the LITO-modified devices still retained 89.6% of their initial efficiency. A linear extrapolation of the test results predicts a *T*_80_ (the time to reach 80% of the initial efficiency) of 8843 h. In the accelerated degradation test, where the devices were placed on a hot plate at 65°C, the *T*_80_ remained at 1296 h. In contrast, the efficiency of PH1000-modified devices decreased rapidly under room temperature conditions, with a *T*_80_ of only 10 h. The lifetime of the PH1000-based devices was extended when heated, with the *T*_80_ increasing to 75 h. This phenomenon may be attributed to the hygroscopic nature of PH1000 [48]. Additional device parameters from the stability tests are provided in Figs S16 and S17. These indicate that PH1000 accelerates the corrosion of metal and the ZnO electron transport layer due to its hygroscopic and acidic properties, which is the reason for poor stability in the ambient environment.

To further evaluate the stability of the AgCu/LITO electrodes and corresponding devices under more stringent environmental conditions, accelerated aging tests were conducted under harsh stress environments. First, the electrical stability of the electrodes was assessed under high-temperature and high-humidity conditions (85°C/85% RH). After 1000 h of aging, the sheet resistance of the electrode increased from approximately 0.8 to around 1.0 Ω/□, while still maintaining acceptable conductivity (Fig. S18). Subsequently, the photostability of 1 cm^2^ devices was investigated under a nitrogen atmosphere via maximum power point (MPP) tracking for nearly 1000 h. The device retained over 80% of its maximum efficiency throughout the test, demonstrating excellent operational stability (Fig. S19). These rigorous stability evaluations indicate that the LITO-modified devices exhibit enhanced resilience under demanding conditions, significantly enhancing their potential for practical application in real-world environments.

In general, commercial flexible ITO-based devices tend to have poor bending tolerance, which limits their further application [49,50]. However, the sputtered LITO material in our study was deposited at low temperatures (as opposed to ITO, which requires heating to enhance crystallization), and it demonstrated superior performance in bending stability tests. The bending performance tests on the transparent conductive substrates were carried out with a bending radius of 5 mm (Fig. 3c). The test method is illustrated in the inset of Fig. 3d, where the substrate was placed tightly against a stainless-steel cylinder with a radius of 5 mm. Downward pressure was applied to bend the device, and this process was repeated multiple times in the same direction. After every 1000 bends, the transmittance and *R*_sh_ of the substrate were measured, and the complete transmittance spectra are shown in Fig. S20. The transmittance of the three substrates is almost unchanged by bending. Compared to transmittance, the *R*_sh_ showed a clear increasing trend with the number of bending cycles. After 1000 bends, the *R*_sh_ of PET/ITO increased by nearly two orders of magnitude to 816 Ω/□. The PH1000-modified substrate showed slight improvement, but by the end of the test, the *R*_sh_ also rose to 48 Ω/□. Notably, the *R*_sh_ of the LITO-modified substrate showed almost no change after 1000 bends. As shown in Fig. 3e and Figs S21 and S22, after bending, PET/ITO exhibits distinct non-periodic vertical cracks, indicating fractures of ITO along grain boundaries. For the PH1000-modified substrate, dark vertical stripes appear at the silver grid locations after bending, suggesting breakage in the silver grid. In contrast, the copper-filled grids in the LITO-modified substrate remain intact, confirming that the composite electrode effectively preserves the flexibility of the substrate. In addition, the mechanical robustness of the electrode was further evaluated through adhesion testing of the LITO layer. According to the ASTM D3359-09 standard, a grid-cutting tool with a 1 mm spacing was used to scribe the surfaces of both PET/LITO and PET/AgCu/LITO samples, followed by tape peel testing. The results are presented in Fig. S23. Optical microscopy analysis revealed only minor film delamination at the grid intersection points in both cases. Nevertheless, small flakes of the film were detached at intersections, achieving a 4B rating (less than 5% affected area) as defined by the ASTM standard.

Although a 30 nm-thick LITO layer does not affect the bending stability of the metal grid electrode, the electrical conductivity of the composite electrode degrades significantly with increasing LITO thickness. As shown in Table S10, when the LITO thickness reaches 60 nm, the *R*_sh_ increases by one order of magnitude after 900 bending cycles. At a thickness of 135 nm, the degradation is even more pronounced, with the sheet resistance rising to 6661 Ω/□ after 1000 bending cycles. These results clearly indicate that LITO thickness critically influences the bending stability of the composite electrode, and an optimal thickness must be balanced between mechanical robustness and electrical performance.

The bending test of single-cell devices was then performed under conditions with a bending radius of 5 mm. The device performance was measured and recorded, and the results are shown in Fig. 3d. After 1000 bends, the LITO-modified device still retained 97% of its initial efficiency, whereas the PH1000-modified device dropped below 80% of its initial efficiency after just 700 bends. The detailed device parameters for the bending tests are shown in Fig. 3f. The *V*_OC_ was generally unaffected by the bending experiments, while the FF showed a steady decline with an increasing number of bends. The increase in series resistance (*R*_s_) was the primary factor affecting the FF (as shown in Fig. 3f). After 700 bends, the *R*_s_ of the PH1000-based device increased from 3.38 to 5.34 Ω, corresponding to a drop in FF to 82.7% of its initial value. Bending tests on the transparent conductive substrates also verified this trend in series resistance.

These results demonstrate that the AgCu/LITO composite electrode not only addresses the issue of PH1000 being susceptible to degradation by water vapor, thereby improving stability under ambient conditions, but also enhances the flexibility of the devices. This improvement is crucial for the practical application of flexible OSC modules in real-world conditions.

### Application of flexible OSC modules in range-extended SUAV systems

**Figure 4.**
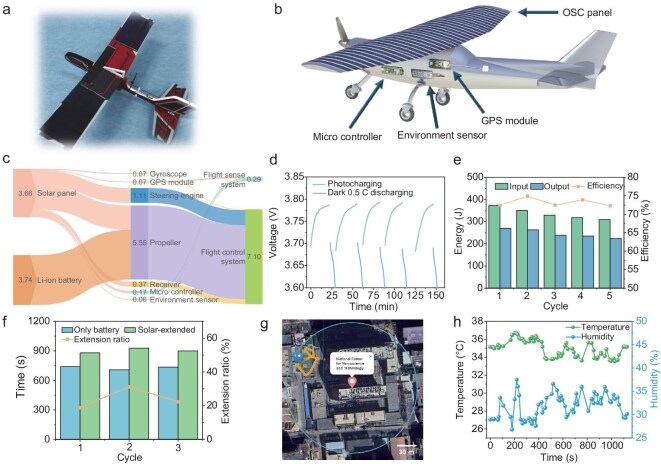
Flexible solar-storage integrated system and range-extended SUAV. (a) Images of the range-extended SUAV. (b) Schematic diagram of SUAV fuselage load modules. (c) Energy flow diagram of SUAV’s takeoff stage. (d) The voltage fluctuation of the battery during the 5-cycle charge–discharge of flexible solar-storage integrated system. (e) Photovoltaic output and lithium battery input energy diagram during the charge–discharge cycle, as well as the cycle efficiency. (f) Comparison of motor endurance time with and without photovoltaic charging. (g) Visualization of the flight trajectory. The scale bar is 30 m. (h) Temperature and humidity sensor data during flight.

We integrated flexible OSC modules onto a small-scale SUAV and combined them with a lithium battery and power management system to extend the flight duration of the drone. Figure 4a shows a small fixed-wing SUAV made of polystyrene, weighing 29 g. The wing surface is covered with six 42 cm^2^ flexible OSC modules. The module was encapsulated using a PET-based thermal sealing method. After encapsulation, the average mass of the module was 1.88 g, corresponding to an average power-to-mass ratio of 339.7 W/kg. Due to the limitation of the SUAV’s maximum takeoff weight, the photovoltaic module does not have an independent thermal control unit to prevent the module temperature from rising during flight. The integrated systems of the SUAV are shown in Fig. 4b. The photovoltaic modules are arranged in two sets of three serially connected panels, with both sets connected in parallel to a MPP tracking charging circuit. The circuit provides two 5 V outputs: one powers the Flight Sense system (which includes a microcontroller, environmental sensors and GPS module) and the other, along with a 3.7 V 380 mAh lithium battery, powers the Flight Control system (which includes the propeller, servos and gyroscope). The total takeoff weight, including all components, is 109 g. For balance purposes, the Flight Sense system is mounted on one side of the fuselage. Table S11 lists the power consumption of the SUAV’s systems, with the engine power estimated based on maximum output. The motor’s maximum output current is 1.5 A, which exceeds the capacity of the photovoltaic modules, thus necessitating a high-discharge-rate lithium battery. As shown in the energy flow diagram in Fig. 4c, the Flight Sense system accounts for 4% of the total power consumption, while the remaining power from the photovoltaic modules, after sustaining the sensing system, is combined with the lithium battery to meet the flight energy requirements.

We then conducted a charge–discharge cycling test on the integrated photovoltaic-lithium battery system in a static environment. Since a full charge–discharge cycle at a 0.5 C rate for the lithium battery takes nearly 4 h (Fig. S24), the cycle test began with the battery voltage at 3.6 V (corresponding to a state of charge, SOC, of approximately 50%). Under AM 1.5G illumination, the solar modules charged the battery for 20 min, followed by discharging at a 0.5 C constant current rate in a dark environment until the voltage returned to 3.6 V. Figure 4d shows the voltage changes during the charging (green line) and discharging (blue line) processes, with the system exhibiting stable performance over five cycles. Figure 4e displays the energy and charging efficiency over five cycles. Due to the presence of a blocking diode in the MPP circuit, which causes voltage drop, the charging efficiency is around 72%. This efficiency loss can be improved by using a higher voltage through serially connected lithium batteries. The UAV endurance test results are presented in Fig. 4f. The test commenced with three fully charged lithium batteries, after which the motor was set to operate at a specific rotational speed until it ceased, marking the end of a test cycle. Multiple trials were conducted both with and without photovoltaic assistance (test data are detailed in Fig. S25). The results demonstrate that under photovoltaic-assisted power supply conditions, the motor’s operational time was extended by an average of 24.2%.

Figure 4g and h presents data collected from the GPS and environmental sensors during a test flight. The GPS data, visualized using Python’s Folium package, illustrates the UAV’s flight path. In Fig. 4g, the red icon marks the main building of the National Center for Nanoscience and Technology, while the blue icon and orange lines represent the flight trajectory, scaled at 300 m. The onboard light sensor and atmospheric sensors (measuring temperature, humidity and pressure) recorded environmental data, shown in Fig. 4h. Throughout the flight test, the onboard equipment functioned as expected, successfully collecting data and validating the feasibility of the SUAV’s integrated system.

## CONCLUSIONS

We achieved the fabrication of high-efficiency flexible OSC modules by optimizing the modification of the metal mesh electrode. The traditional PH1000 conductive polymer was replaced with the metal oxide LITO, and low-temperature sputtering processes were employed to address the bending brittleness of the metal oxide. In bending tests with a radius of 5 mm, after 1000 cycles of bending, the LITO-modified devices maintained 97% of their initial efficiency, while the efficiency of PH1000-based devices dropped to below 80%. We also tested the stability of the devices based on the ISOS protocol; in ambient conditions, the unencapsulated LITO devices retained 89.6% efficiency after being placed at room temperature for 1080 h, and under 65°C heating tests, the *T*_80_ was also greater than 1000 h, demonstrating excellent device stability.

By introducing LITO to smooth the surface of the AgCu mesh and effectively improving charge transport in the composite electrode without sacrificing transparency, the *FoM* of the substrates more than doubled compared to Ag/PH1000 ones. Consequently, the efficiency of the 1 cm^2^ single-cell devices reached 17.12% (16.88% certified PCE), with a 42 cm^2^ module PCE of 15.60%, both representing some of the highest values for non-halogen solvent flexible OSCs.

Finally, we applied the flexible OSC modules to an SUAV, integrating them with lithium batteries to form a solar-storage system. The solar-storage system exhibited stable performance during multiple charge–discharge cycles. The motor endurance test results indicate that under photovoltaic-assisted conditions, the working time can be extended by 24.2%. During flight, the system successfully collected and transmitted flight sensor data, validating the feasibility of using OSC-extended drones for environmental monitoring and remote sensing applications.

We demonstrate the practical significance of flexible OSC modules in lightweight mobile systems, especially in scenarios requiring extended off-grid operation. Looking ahead, further improvements in OSC efficiency and durability, along with optimizations in power management systems, will expand their application potential in aerospace, wearable electronics and other emerging technologies. By overcoming current limitations, OSCs could play a crucial role in future renewable energy solutions.

## MATERIALS AND METHODS

All experimental details and methods are provided in the Supplementary data.

## Supplementary Material

nwaf519_Supplemental_File
