# Supplementary material for: High-efficiency and flexible organic solar modules with promising applications in solar-extended unmanned aerial vehicles
Source: Natl Sci Rev. 2025 Nov 19;13(1):nwaf519. doi: 10.1093/nsr/nwaf519 (PMC12796806; doi:10.1093/nsr/nwaf519)
Supplement: nwaf519_Supplemental_File [file nwaf519_supplemental_file.pdf]

Supporting Information

High-efficient and flexible organic solar modules with promising applications in solar-extended unmanned aerial vehicles

Chenyang Tian,[1,3,†] Hao Zhang,[1,†] Ziqi Zhang,[2] Caixuan Wang,[1,3] Dan Deng,[1] Dingding Qiu,[1] Jing Tao,[1,3] Kamran Amin,[1] Kaiwu Peng,[1] Jia Li,[2] Tong Wang,[1] Yuhan Wang,[1,3] Jianqi Zhang,[1,*] and Zhixiang Wei[1,3,*]

[1]CAS Key Laboratory of Nanosystem and Hierarchical Fabrication, National Center for Nanoscience and Technology, Chinese Academy of Sciences; Beijing 100190, China

[2]Ningbo Institute of Materials Technology and Engineering, Chinese Academy of Sciences; Ningbo 315201, China

[3]University of Chinese Academy of Sciences; Beijing 100049, China

*Corresponding authors. weizx@nanoctr.cn, zhangjq@nanoctr.cn

[†]Equally contributed to this work.

MATERIALS AND METHODS

Materials

PM6 used in this work was purchased from Hyper, Inc. (China). N3, was obtained from eFlexPV Inc. Trimer, ZnO nanoparticles and NMA were synthesized according to the published articles[1, 2]. 1,8-Diiodobenzene (DIB) was purchased from Tokyo Chemical Industry Co., Ltd. The solvent o-xylene was purchased from Sigma-Aldrich. PEDOT:PSS(PH1000) aqueous solution was purchased from Heraeus. Evaporated silver was purchased from ZhongNuo Advanced Material Technology. MoO_3 (99.99%) was purchased from Strem Chemicals, Inc. The PET substrate deposited with ITO was purchased from Youxuan New Energy Technology Co., Ltd. The PET substrates with printed silver grid and silver-copper grid used for manufacturing large-area devices were purchased from Suzhou NanoGrid Technology Co., Ltd.

Fabrication of large area devices and measurement of device efficiency

For all flexible OSC devices, the inverted configuration (PET/Metal-grid/Modified Layer/ZnO+NMA/Active layer/MoO_x/Ag) was utilized. Patterned metal-grids were embedded into PET substrates. Mini-roll Coater (FOM Technologies, Denmark) was utilized as the R2R SD coater. All the solution procedures were conducted in an ambient atmosphere. For PH1000 modified devices, PEDOT:PSS aqueous solution was diluted with isopropanol at a ratio of 1:4 (v/v), then slot-die coated on the substrates with an injection speed of 3.5 μL/s and coating speed of 10 mm/s. After that, the hybrid electrodes were thermally annealed at 120 °C for 20 min in ambient air. The ZnO nanoparticles were dispersed in isopropanol (20 mg/mL), NMA at a

concentration of 1.5 mg/ml was added and stirred, injected at a speed of 1.2 μL/s with a coating speed of 10 mm/s. The donor and acceptors were dissolved in *o*-xylene with total concentration of 21.2 mg/mL (PM6:Trimer:N3 = 1:1.5:0.15 w/w for ternary blend) and 2 mg/mL DIB was added as the solid additive. After heating the roller to selected temperature, the active layer was slot-die coated with an injection speed of 1.7 μL/s and coating speed of 13 mm/s. During the coating process, the slot-die head and solution were brought to the experimental set temperature using a homemade heating module. In all coating sessions, the gap between slot-die head and the substrate is controlled at 125 ± 10 μm by using a contact sensor to calibrate servo motors. The strip width of slot-die head is 13 mm. 10 nm MoO_x layer and 160 nm Ag electrode were thermally evaporated under a pressure of 1.0×10^{-4} Pa. For both single cells and the modules, the length and width of each sub-cell were determined by using different evaporation masks.

For LITO modified devices, patterned silver-copper composite metal grid was printed on PET substrates. The LITO deposition was assisted by Ningbo Najin Film Co., Ltd. in the following procedures: Place the transparent substrate into vacuum chamber of the roll-to-roll sputtering machine (R2R sputtering system SF-R600), and the substrate was plasma treated with argon gas. The pressure of gas was controlled to 2×10^{-3} mbar and applied voltage was 1000 V. The working atmosphere of magnetron sputtering process was argon and oxygen, with an oxygen ratio of 5% and a pressure of 0.35 Pa. The sputtering target was an indium tin alloy, and the target power was 4 kW. The final modified layer thickness varied with deposition time. The subsequent fabrication steps were consistent with the PH1000 modified devices.

The flexible OSC module was fabricated by connecting individual sub-cells in a series connection.

Each sub-cell was fabricated using the same device configuration and processing protocol as described previously. The interconnection between adjacent sub-cells was achieved through a three-step process: (a) pre-patterned transparent electrodes (P1 process), (b) sequential slot-die coating deposition (P2 process), and (c) mask-defined evaporation of the top electrode (P3 process). The active area of each sub-cell was defined as 1 cm in width, with a 0.3 cm-wide dead zone between adjacent sub-cells.

The *J–V* measurement was performed under AM 1.5G (100 mW/cm^2) with a SAN-EI ELECTRIC XES-1004SE-200S solar simulator. Light intensity was calibrated by a Newport Oriel 91150V-KG5 Si-based solar cell. *J-V* characteristics were recorded with a Keithley 2400 SourceMeter. The cells were measured in air at the temperature of 25 °C, scanning from -0.2 V to 1 V and dwell time of 1 ms for every point. The mask area is 1.0 cm^2. The EQE spectrum was measured through the Solar Cell Spectral Response Measurement System FETOS-QE-3011 (Enli Technology Co., Ltd., Taiwan). The 1.0 cm^2 flexible OSC was certified in National Photovoltaic Product Quality Inspection & Testing Center (Chengdu) under a large-area solar simulator (Apollo-ss2622-AAA) using a Keithley 2430 SourceMeter in air at a temperature of 25 °C and humidity of 50%.

UV-vis spectroscopy and film uniformity characterization

The film spectra were collected by a Filmetrics F20 spectrometer in the wavelength range of 380–1050 nm. The integration time of the spectrometer was set to 20 ms. For film uniformity characterization, the spectral characterization of the continuously coated films was carried out on a self-built device with a combination of the spectrometer and the motion controller. The detector

and light source are fixed above and below the motion stage, respectively. The film was fixed and passed through the detector with a horizontal sampling interval of 1.0 cm and a vertical sampling interval of 1.3 cm.

SEM and FIB-SEM characterization

SEM SU8200 model was used to get SEM images. The ZnO/NMA samples were sputter-coated platinum layer to improve conductivity. Imaging was conducted under high vacuum conditions at an accelerating voltage of 7 kV and a working distance of 10 mm. The electron beam current was set to 10 μA. For FIB-SEM characterization, all samples were performed by FEI Nova 200 NanoLab dual-beam FIB system, with the incident ion beam perpendicular to the sample surface. In each experiment, 30 keV Ga ions were delivered to the sample surface with beam current of 7 nA for rough milling and 3 nA for final polishing. The collected 2D images were processed and aligned using ImageJ.

Sheet resistance tests of different composite electrodes

Sheet resistance was measured by digital four-probe system (RTS-9, Guangzhou FourProbes Co., Ltd.).

Fabrication of flexible solar-storage integrated system and SUAV and characterization of the charge-discharge cycling performance

Flexible solar-storage integrated system consisted of a flexible OSC module, a lithium battery (3.7V, 380 mAh, 20C), and an MPP tracking power management circuit board (Solar Power Manager, WaveShare Co., Ltd.). These components were connected in series using external wires.

The drone was homemade using KT boards as the material. The engine was a 8520 brushed motor, paired with a 75 mm propeller. The flight sensing system included a microcontroller (Raspberry Pico W), an integrated environmental sensor (Environment Sensor HAT, WaveShare Co., Ltd.), a GPS signal receiver (Pico-GPS-L76B, WaveShare Co., Ltd.), and an antenna.

For the characterization of charge–discharge cycling performance, all tests were conducted under standard laboratory conditions (25 °C, 40 %RH). The experimental setup consisted of the following components: flexible OSC modules integrated on the aircraft, connected to a lithium battery (370 mAh, nominal voltage 3.7 V) via a MPP tracking controller. The battery was protected by a battery management system (BMS) with a voltage operating range of 3.0-4.2 V. The data acquisition system comprised a source measure unit (SMU, Precise S100) and an INA219 power monitor. A precision shunt resistor (0.1 Ω, 1% tolerance) was employed for current sensing, enabling a current resolution of 0.1 mA. The S100 SMU was interfaced via the WebSocket protocol, while the INA219 was connected through the I2C bus, enabling synchronized real-time monitoring of voltage and current throughout the cycling tests.

Supplementary Note 1: To evaluate the compatibility of transparent electrodes, the figure of merit (*FoM*) can be derived according to the following equation[1]:

$$T=\left(1+\frac{Z_0\sigma_{\mathrm{Op}}(\lambda)}{2R_{\mathrm{sh}}\sigma_{\mathrm{DC}}}\right)^{-2}$$

where Z_0 is the impedance of free space (Z_0 = 377 Ω), σ_{DC} and $\sigma_{Op}(\lambda)$ are the direct current and optical conductivities of the materials, respectively. The *FoM* is specifically defined as the value of $\sigma_{DC}/\sigma_{Op}(\lambda)$. The wavelength λ corresponding to the transmittance of the transparent electrode is

set as 625 nm in this work. The underlying principle is that a high *FoM* indicates a material that simultaneously exhibits strong electrical conduction (high σ_{DC}) and minimal optical absorption (low $\sigma_{Op}(\lambda)$), which is critical for achieving high transparency and low sheet resistance.

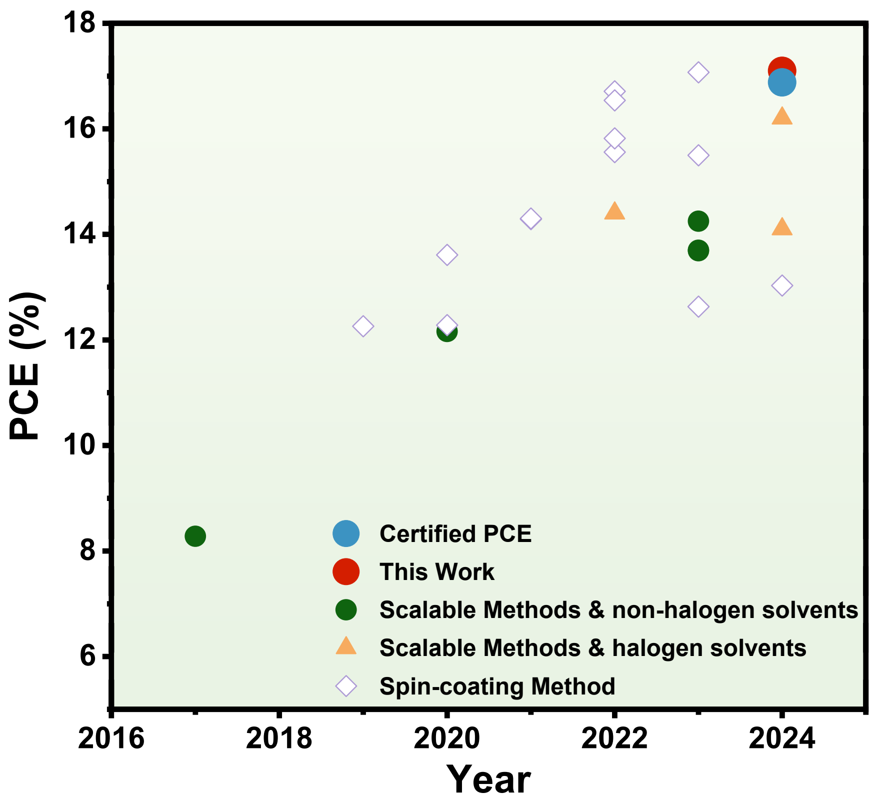

Figure S1. The statistic PCEs of 1 cm^2 flexible OSCs in published articles. Different processing methods and solvents were indicated by various types of data points.

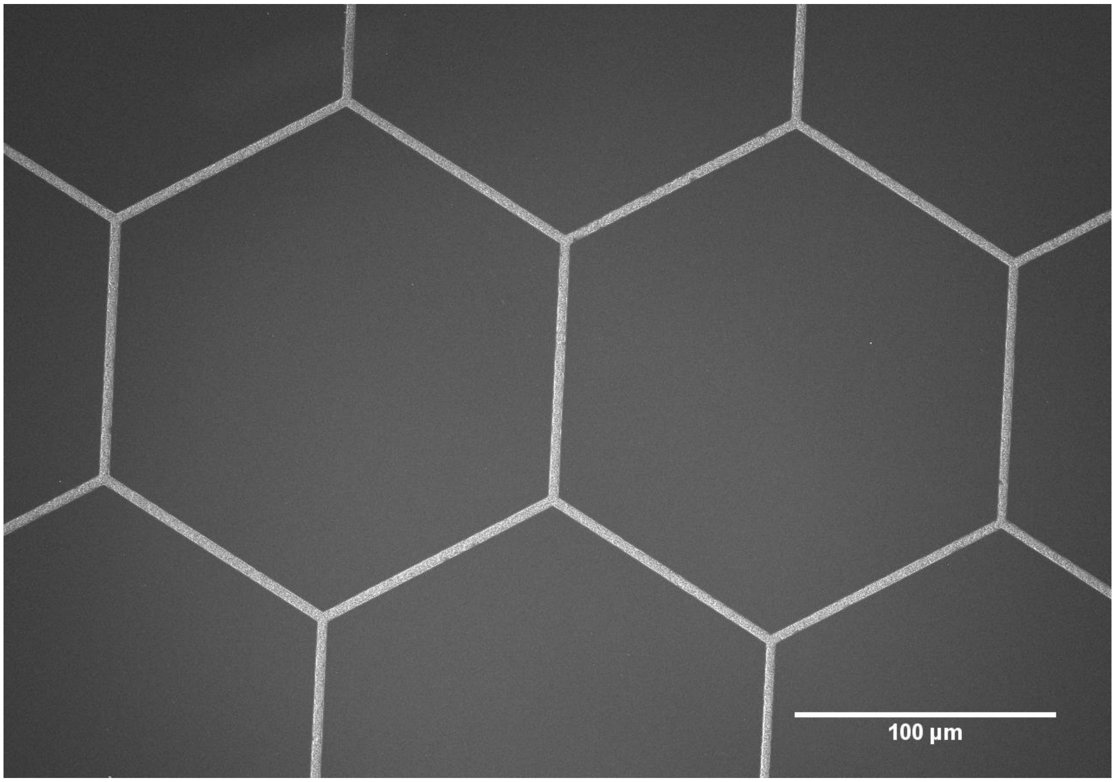

Figure S2. SEM image of metal-grid substrate. The length of hexagon is 100 μm.

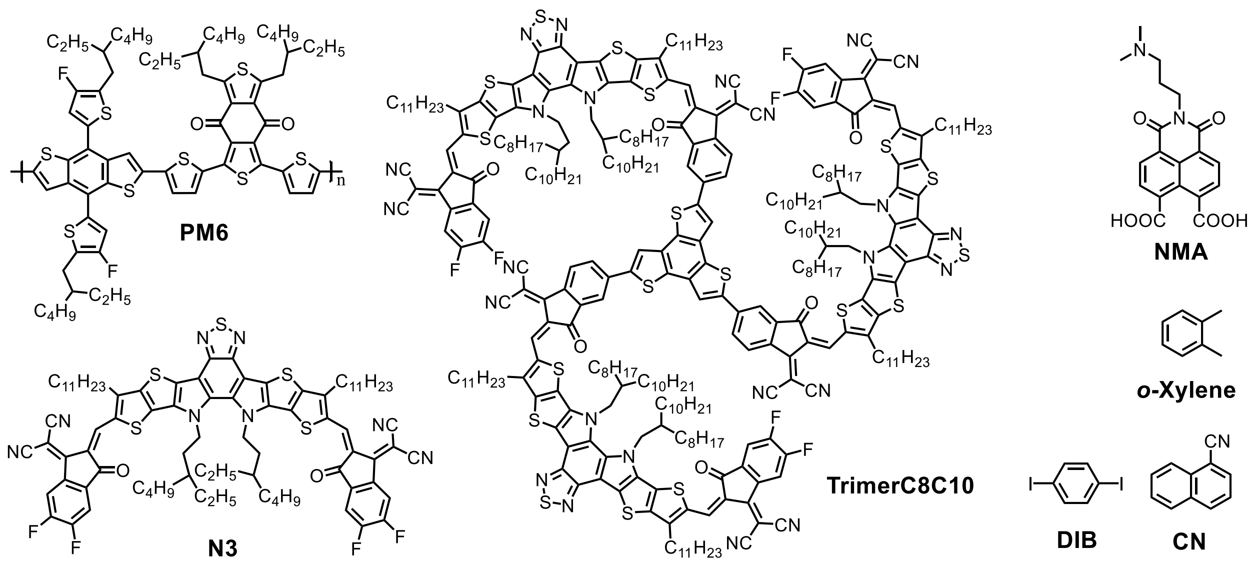

Figure S3. Molecular structures of organic compounds mentioned in the article.

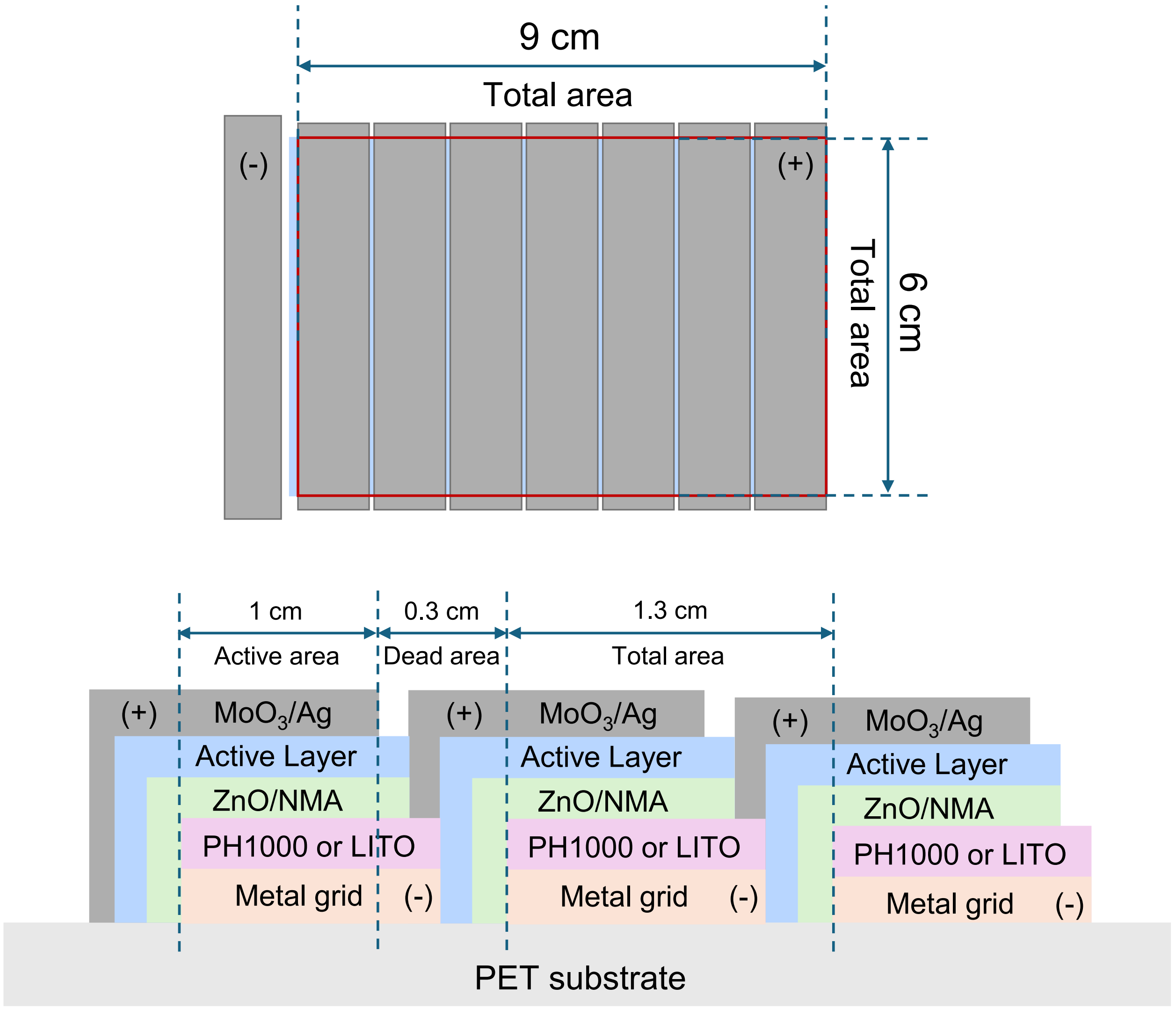

Figure S4. Schematic diagram of the module structure. The top is overall layout of the module, and the bottom is a schematic illustration of the series connection configuration within the module.

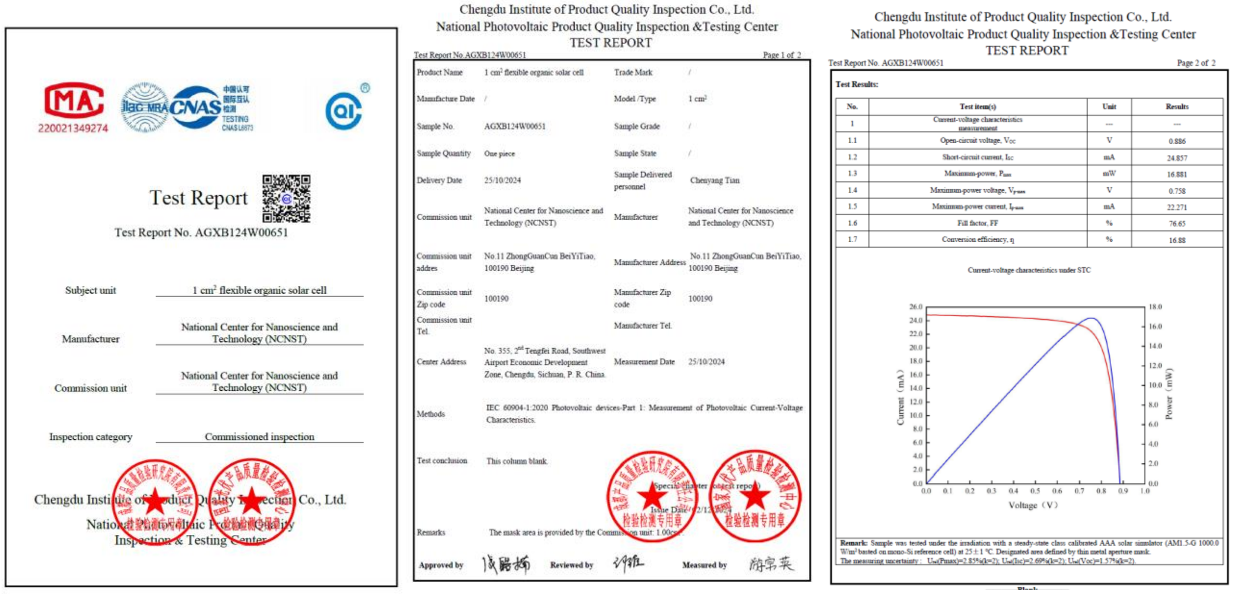

220021349274

Test Report

Test Report No. AGXB124W00651

Subject unit	1 cm^2 flexible organic solar cell
Manufacturer	National Center for Nanoscience and Technology (NCNST)
Commission unit	National Center for Nanoscience and Technology (NCNST)
Inspection category	Commissioned inspection

Chengdu Institute of Product Quality Inspection Co., Ltd.

National Photovoltaic Product Quality Inspection & Testing Center

Chengdu Institute of Product Quality Inspection Co., Ltd.

National Photovoltaic Product Quality Inspection &Testing Center

TEST REPORT

Test Report No.AGXB124W00651 Page 1 of 2

Product Name	1 cm^2 flexible organic solar cell	Trade Mark	/
Manufacture Date	/	Model /Type	1 cm^2
Sample No.	AGXB124W00651	Sample Grade	/
Sample Quantity	One piece	Sample State	/
Delivery Date	25/10/2024	Sample Delivered personnel	Chenyang Tian
Commission unit	National Center for Nanoscience and Technology (NCNST)	Manufacturer	National Center for Nanoscience and Technology (NCNST)
Commission unit addres	No.11 ZhongGuanCun BeiYiTiao, 100190 Beijing	Manufacturer Address	No.11 ZhongGuanCun BeiYiTiao, 100190 Beijing
Commission unit Zip code	100190	Manufacturer Zip code	100190
Commission unit Tel.		Manufacturer Tel.	
Center Address	No. 355, 2nd Tengfei Road, Southwest Airport Economic Development Zone, Chengdu, Sichuan, P. R. China.	Measurement Date	25/10/2024
Methods	IEC 60904-1:2020 Photovoltaic devices-Part 1: Measurement of Photovoltaic Current-Voltage Characteristics.		
Test conclusion	This column blank. Special [illegible] Issue Date [illegible]		
Remarks	The mask area is provided by the Commission unit: 1.00cm^2.		

Approved by [signature] Reviewed by [signature] Measured by [signature]

Chengdu Institute of Product Quality Inspection Co., Ltd.

National Photovoltaic Product Quality Inspection &Testing Center

TEST REPORT

Test Report No. AGXB124W00651 Page 2 of 2

Test Results:

No.	Test item(s)	Unit	Results
1	Current-voltage characteristics measurement	---	---
1.1	Open-circuit voltage, V_{OC}	V	0.886
1.2	Short-circuit current, I_{SC}	mA	24.857
1.3	Maximum-power, P_{max}	mW	16.881
1.4	Maximum-power voltage, V_{P-max}	V	0.758
1.5	Maximum-power current, I_{P-max}	mA	22.271
1.6	Fill factor, FF	%	76.65
1.7	Conversion efficiency, η	%	16.88

Remark: Sample was tested under the irradiation with a steady-state class calibrated AAA solar simulator (AM1.5-G 1000.0 W/m^2 basted on mono-Si reference cell) at 25±1 °C. Designated area defined by thin metal aperture mask.

The measuring uncertainty: $U_{rel}(Pmax)=2.85\%(k=2)$; $U_{rel}(Isc)=2.69\%(k=2)$; $U_{rel}(Voc)=1.57\%(k=2)$.

Figure S5. Third-party certification report of a 1 cm^2 flexible OSC. The device parameters were tested by National Photovoltaic Product Quality Inspection & Testing Center (Chengdu).

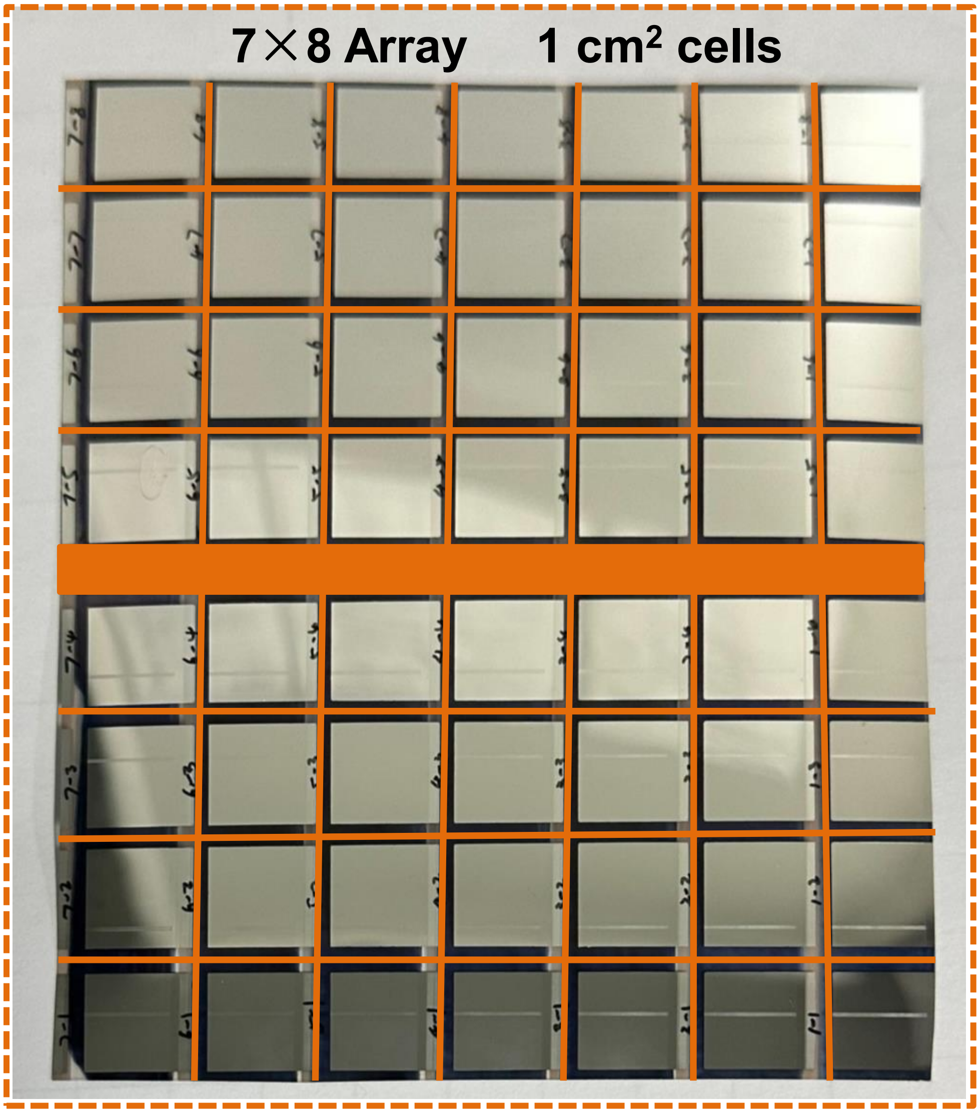

Figure S6. The photograph of flexible 1 cm^2 cells 7×8 array on one substrate.

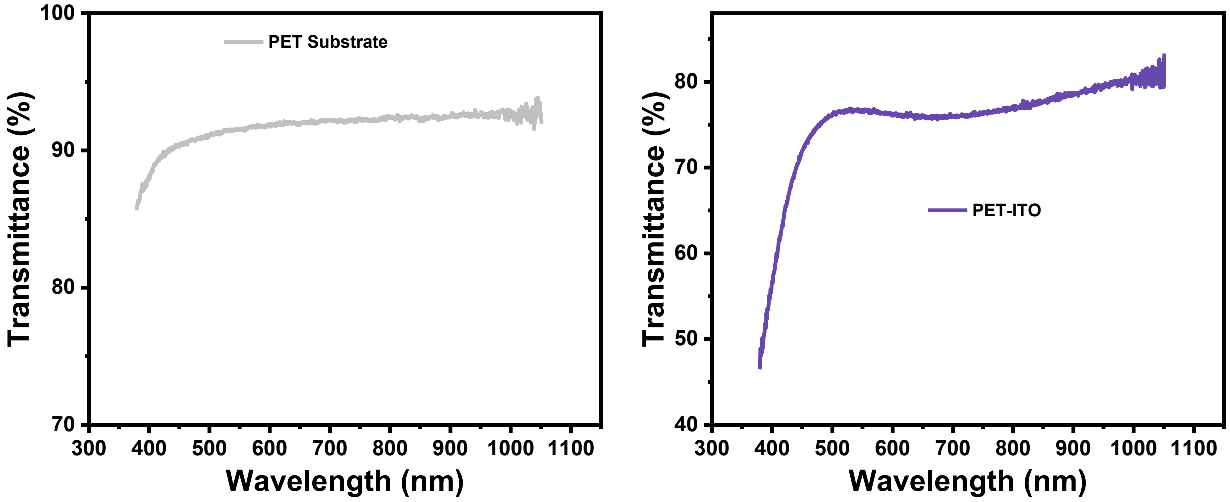

Figure S7. The transmittance spectra of PET and PET-ITO substrates.

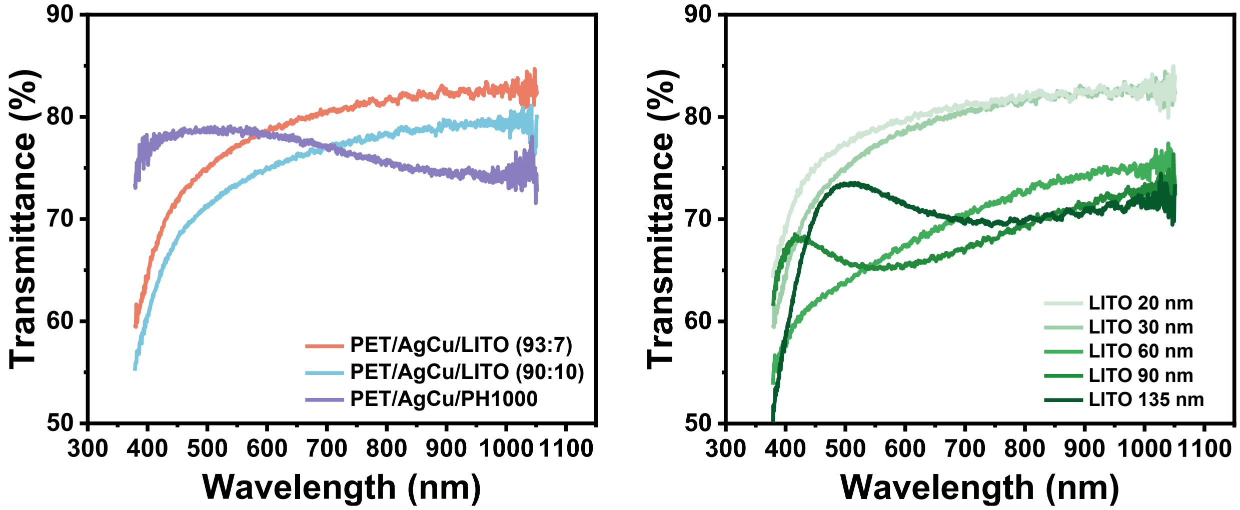

Figure S8. The transmittance spectra of different modification layers on PET/AgCu substrates.

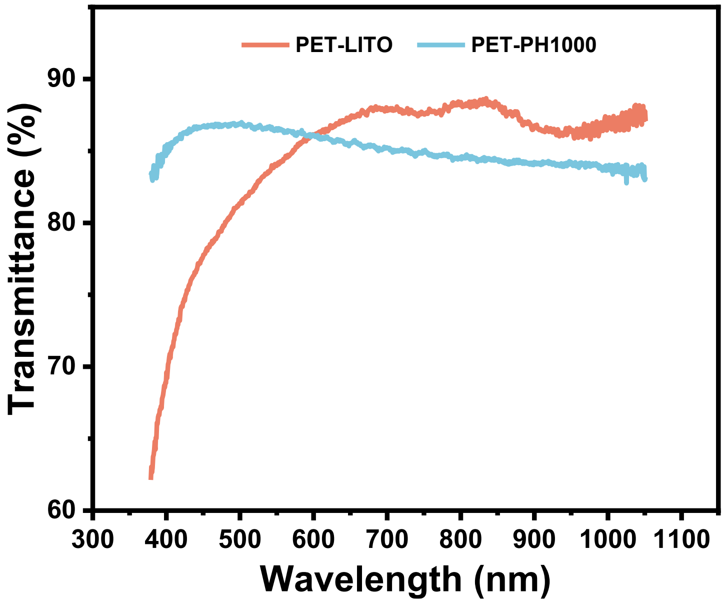

Figure S9. The transmittance spectra of different modification layers on PET substrates.

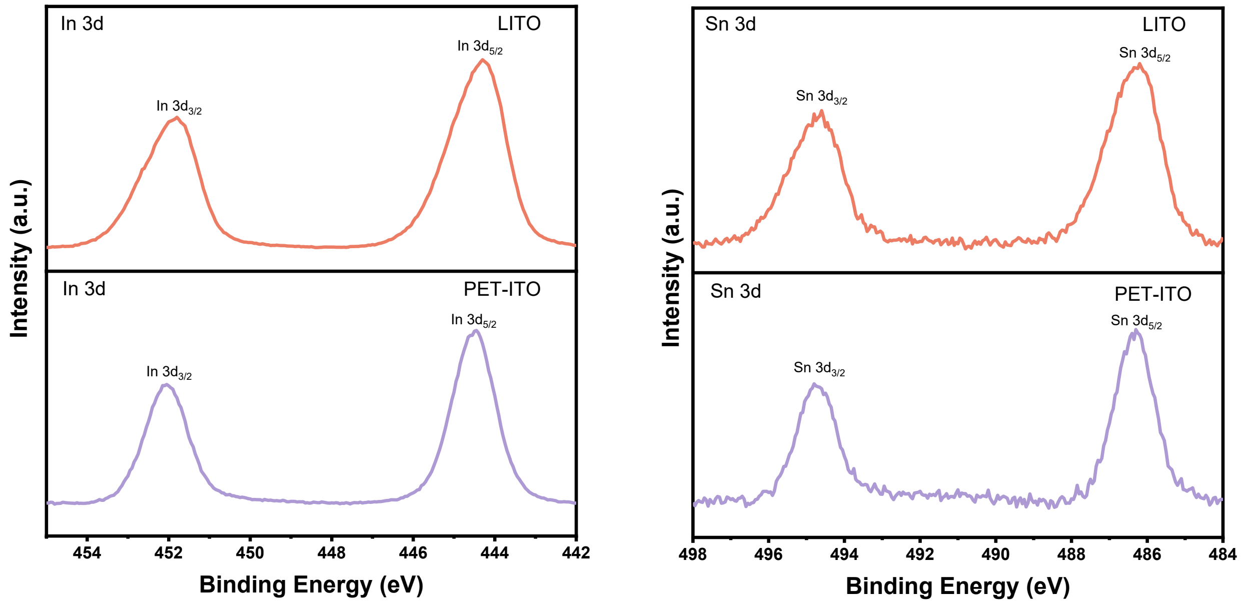

Figure S10. XPS spectra of LITO and PET-ITO for In 3d and Sn 3d core level.

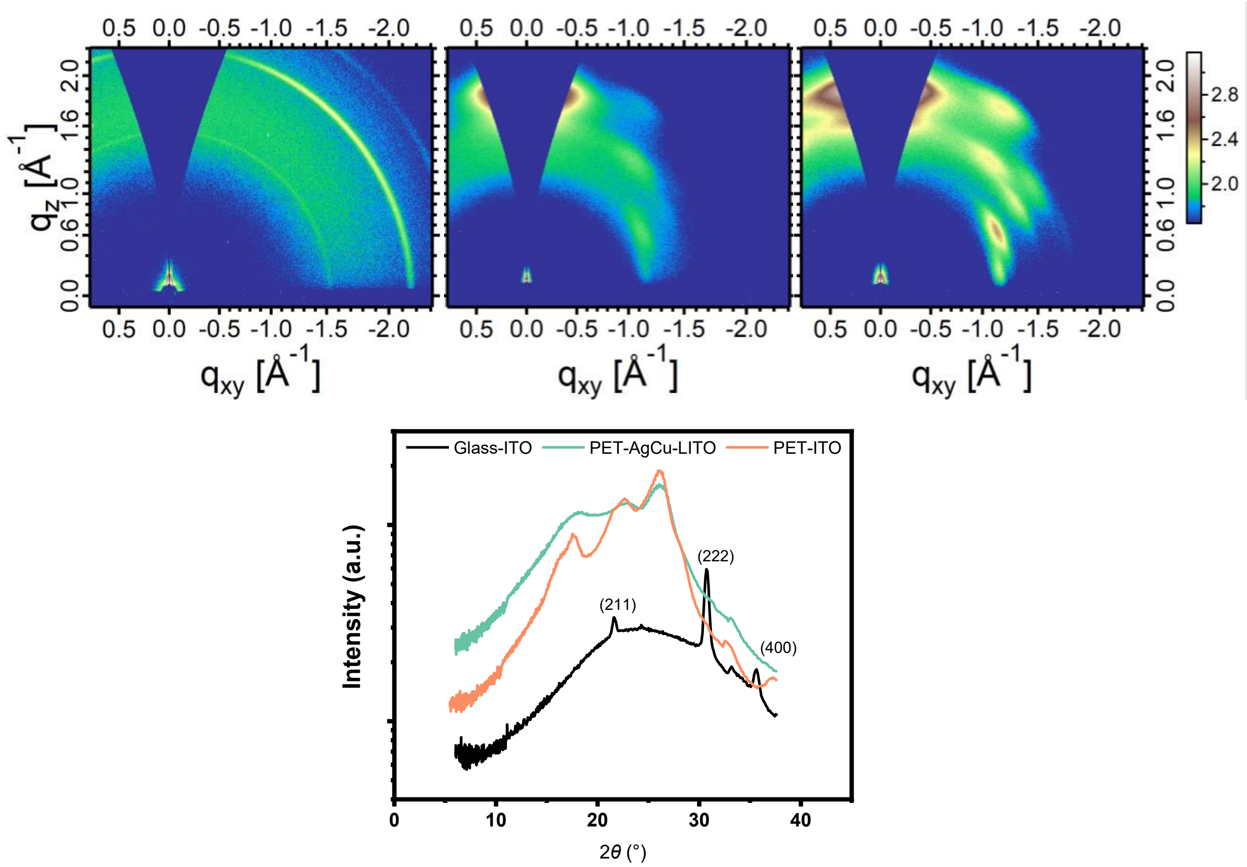

Figure S11. 2D GIWAXS images and diffraction intensity of three types of flexible transparent electrodes. From left to right, the images corresponded to Glass ITO, PET-AgCu-LITO, and PET-ITO. The bottom row showed the plot of diffraction peak intensity versus diffraction angle.

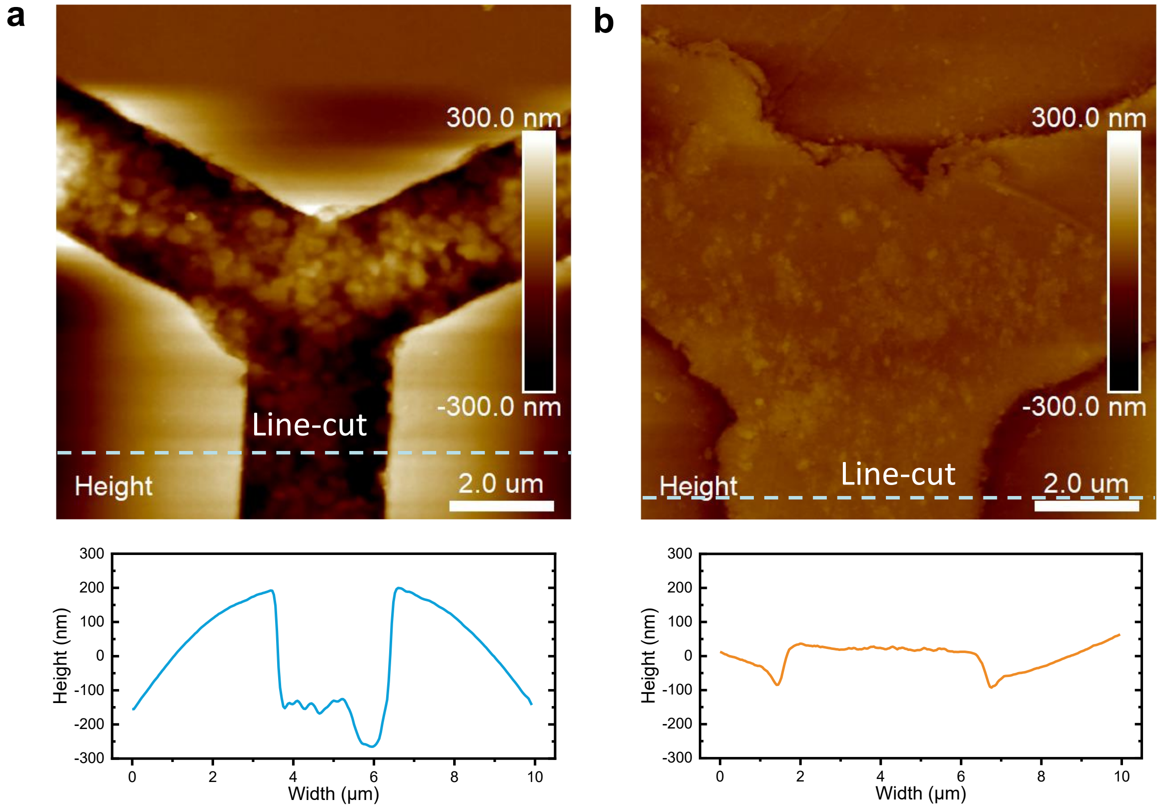

Figure S12. AFM surface morphology and corresponding line-cut height profiles of (a) Ag/PH1000 and (b) AgCu/LITO electrodes.

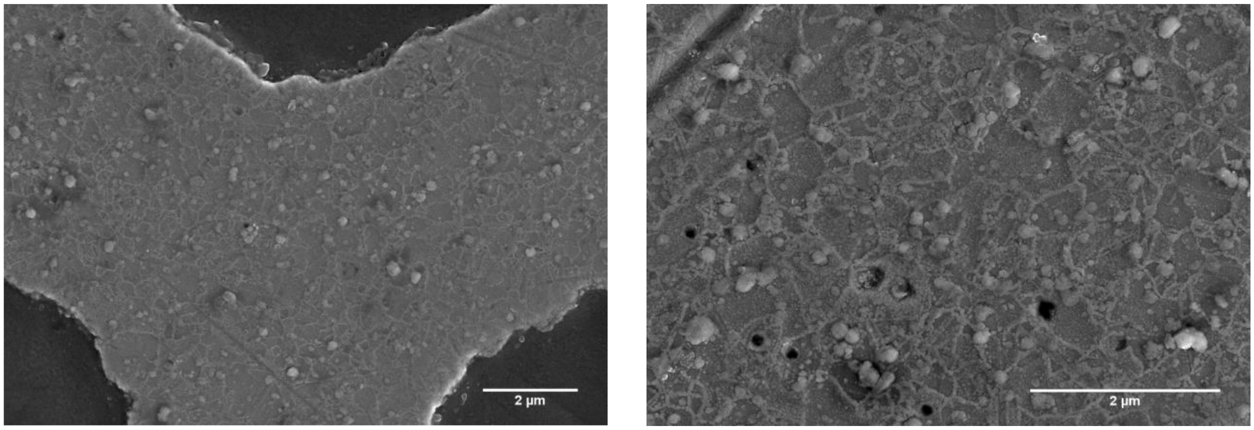

Figure S13. The SEM images of Ag/PH1000 grids. Right picture was taken at high magnification.

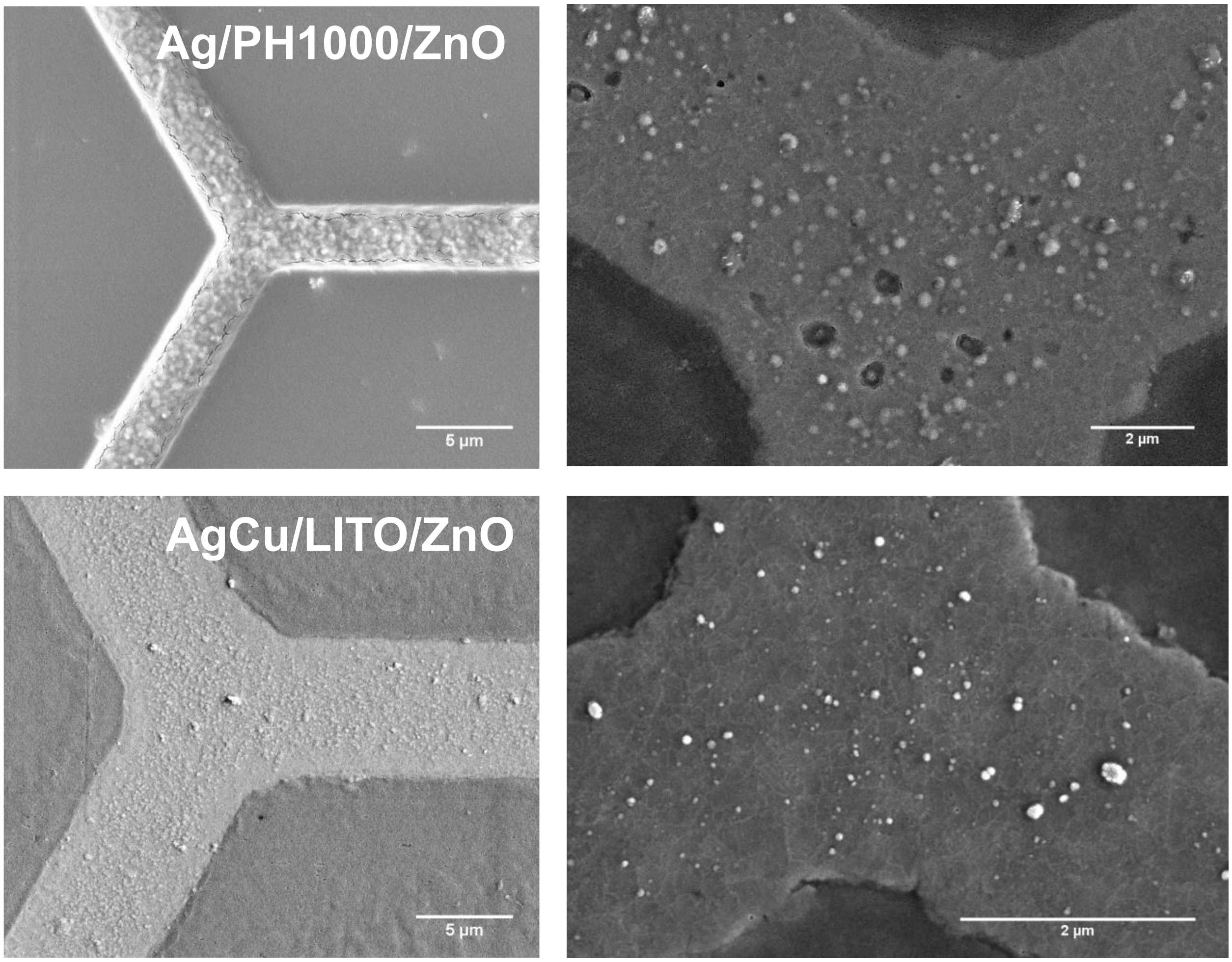

Figure S14. The SEM images of grids coated with ZnO/NMA. The top line was based on Ag/PH1000. The bottom pictures were based on AgCu/LITO. The images in the left and right columns were captured at different magnifications.

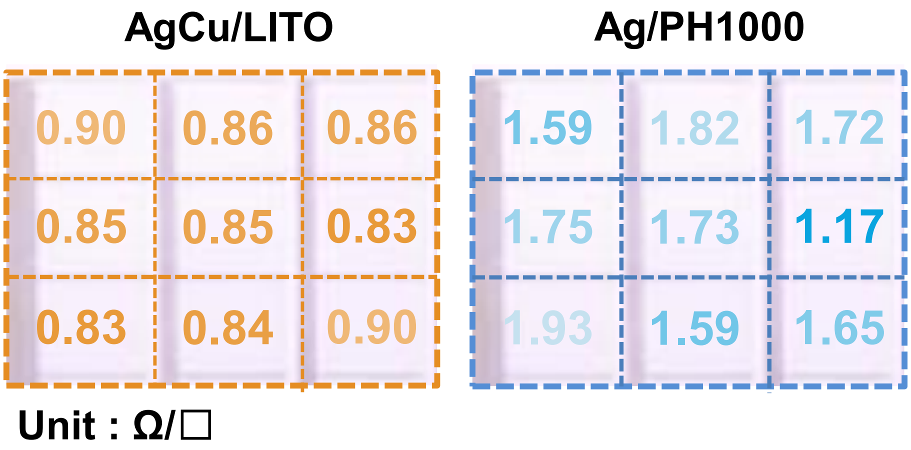

Figure S15. Statistics of sheet resistance of 9 regions of TCEs. The font color from dark to light corresponds to sheet resistance from small to large.

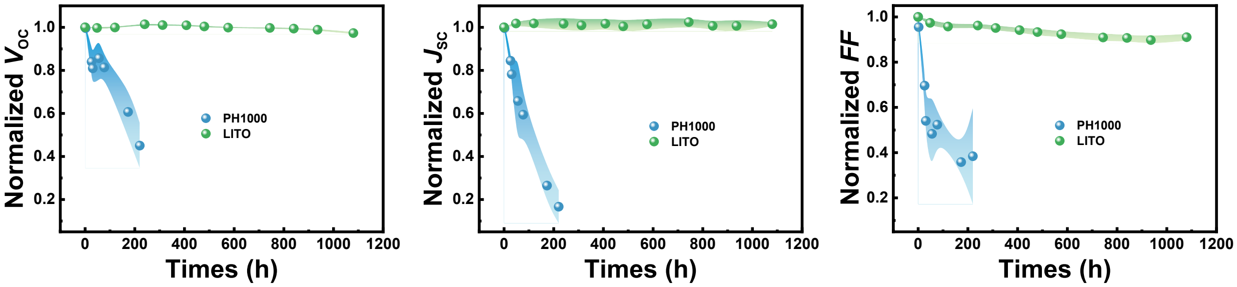

Figure S16. The normalized parameters of devices under ISOS-D-1 stability test.

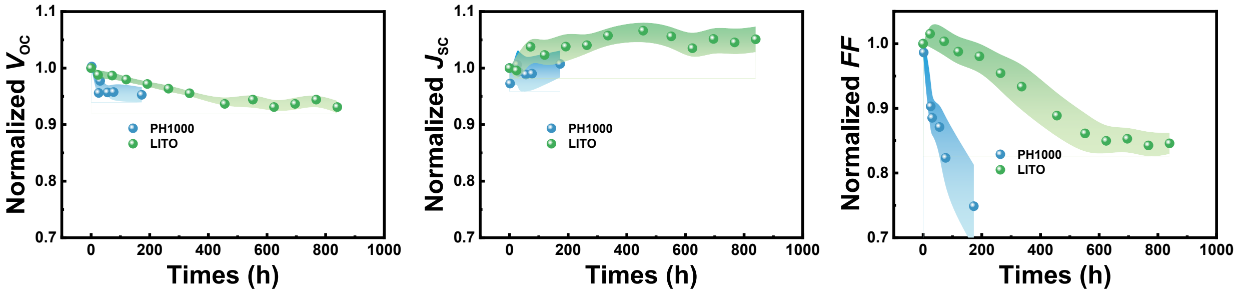

Figure S17. The normalized parameters of devices under ISOS-D-2 stability test.

.

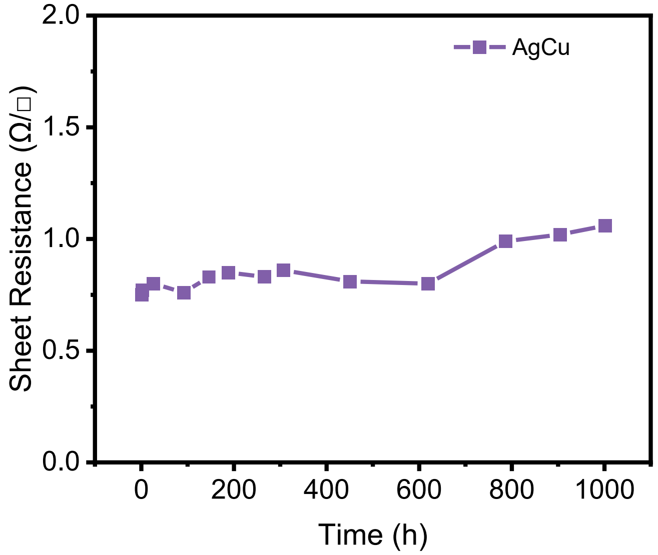

Figure S18. Degradation of sheet resistance of the AgCu/LITO electrode under accelerated aging conditions (85 °C/85 %RH).

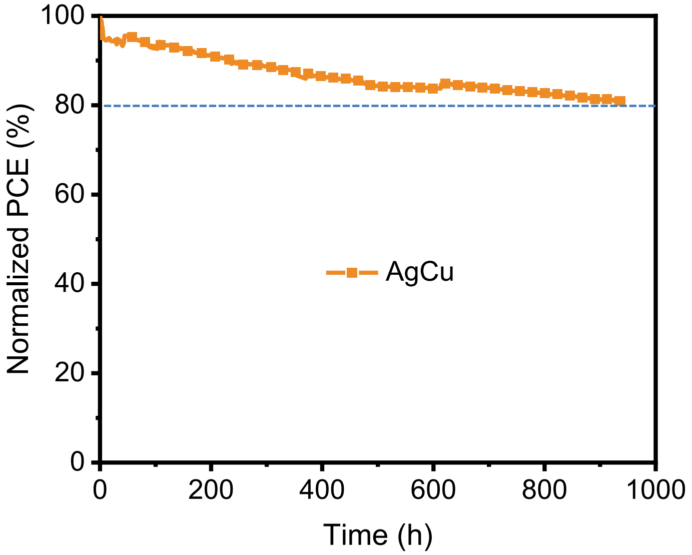

Figure S19. Long-term photostability of a 1 cm^2 device under MPP tracking in nitrogen atmosphere.

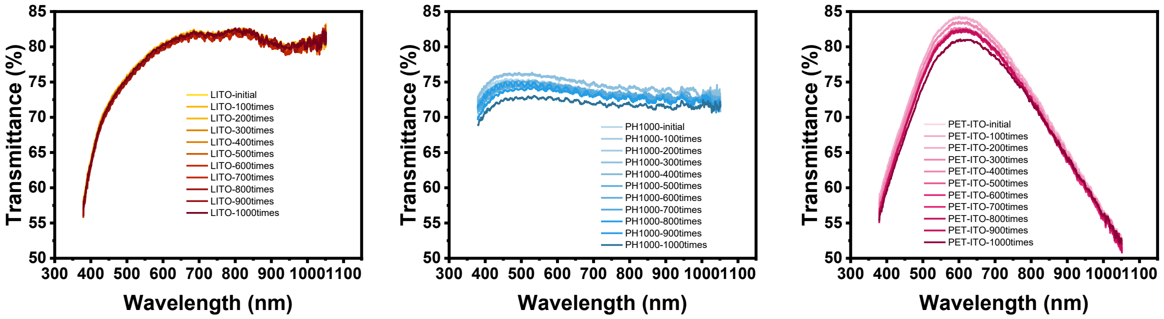

Figure S20. The transmittance spectra of three types of transparent conductive substrates after bending test. (from left to right: AgCu/LITO, Ag/PH1000, ITO).

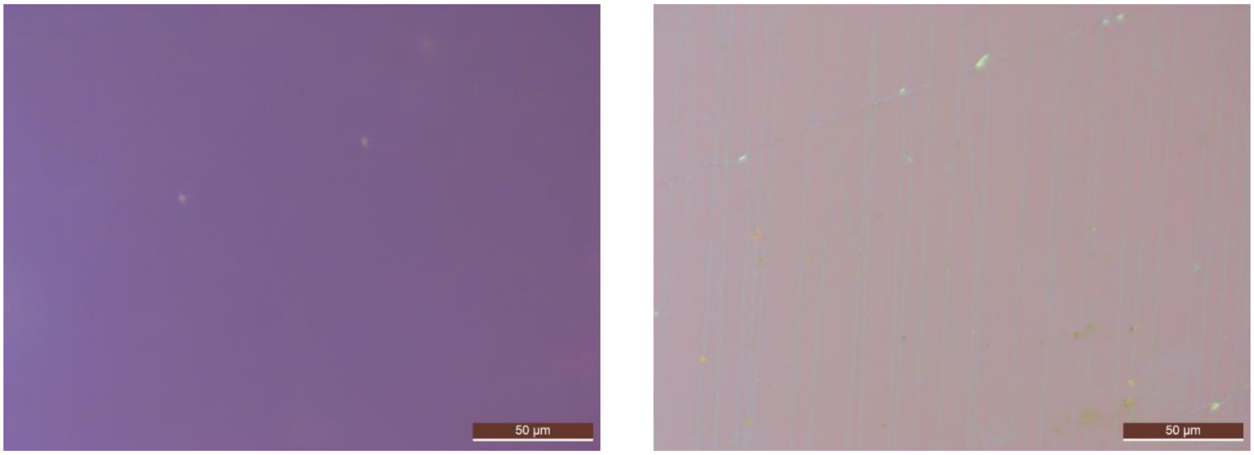

Figure S21. The optical microscope images of PET-ITO substrates. The left column was captured before bending test. The right column was taken after bending test.

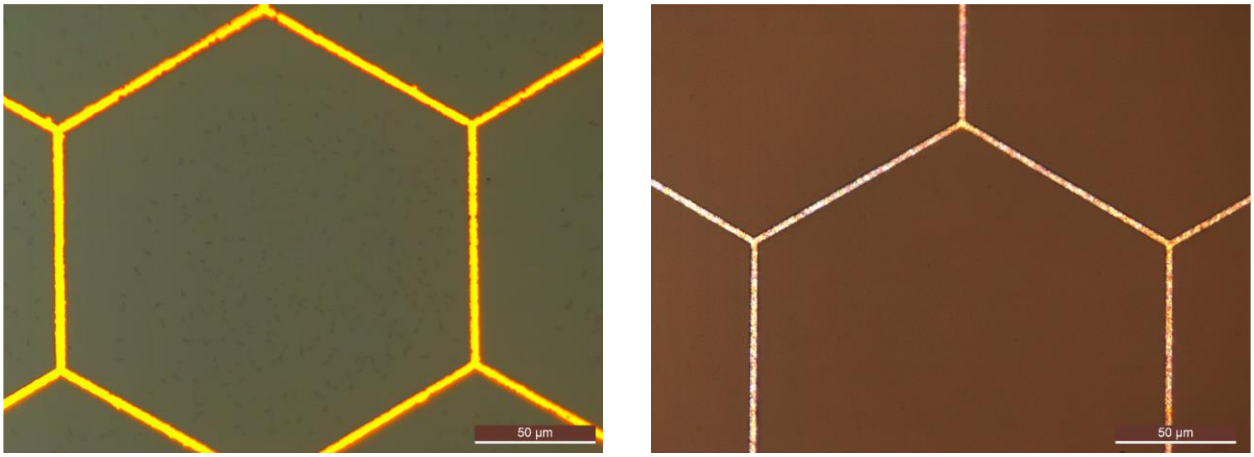

Figure S22. The optical microscope images of AgCu/LITO and Ag/PH1000 substrates. Both images were captured before bending test. The left one was AgCu/LITO and the right one was Ag/PH1000.

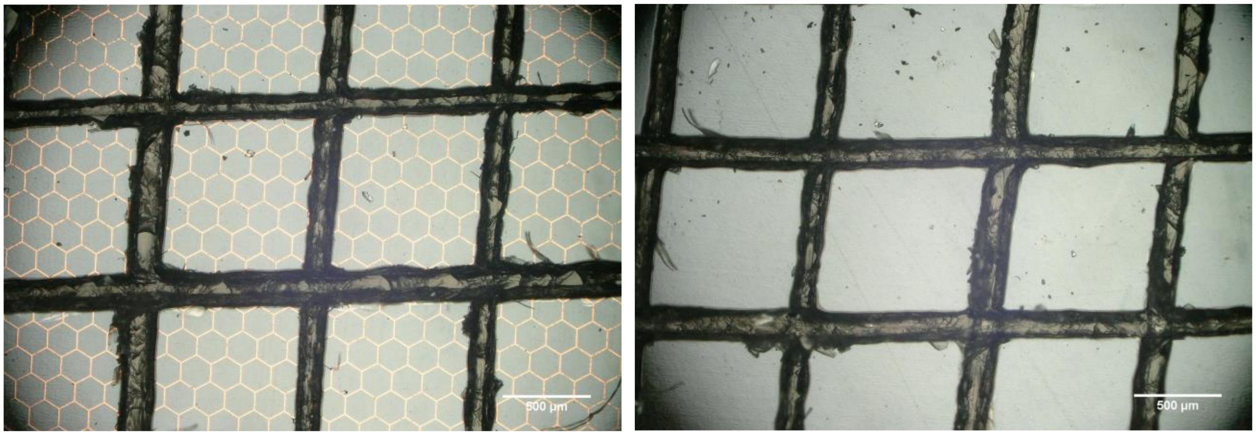

Figure S23. Adhesion test results of transparent electrodes evaluated according to the ASTM D3359-09 standard. The left panel showed PET/AgCu/LITO, and the right panel showed PET/LITO.

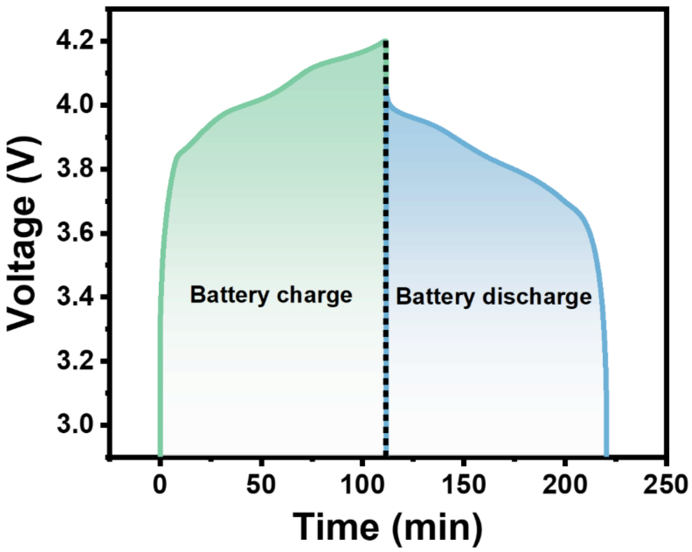

Figure S24. Lithium battery charge-discharge test. The charge and discharge rate was 0.5C.

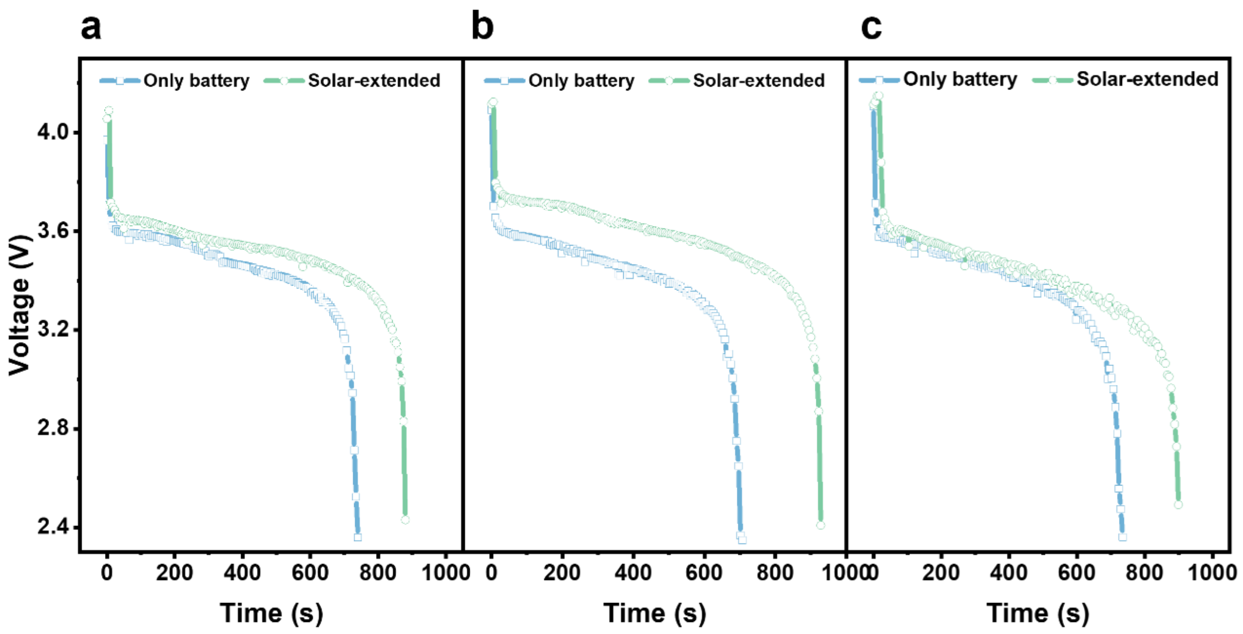

Figure S25. Flight endurance test. The variation of the lithium battery voltage over time during the motor operation was recorded, and the working duration under photovoltaic-assisted charging conditions was compared. The results of three tests are represented as a, b, and c, respectively.

Table S1. The PCE statistical data based on different ratio of third component.

N3 ratio	V_{OC} (V)	J_{SC} (mA/cm^2)	FF (%)	PCE (%)
0	0.880	21.95	74.93	14.48
	0.890±0.008	21.36±0.44	75.51±0.85	14.36±0.06
5%	0.883	21.79	75.67	14.57
	0.881±0.001	21.91±0.13	74.64±0.55	14.41±0.07
10%	0.878	22.35	75.05	14.74
	0.873±0.003	22.41±0.33	73.26±1.18	14.33±0.18
15%	0.880	22.16	74.73	14.58
	0.873±0.006	22.18±0.28	73.19±0.97	14.17±0.21

The data were collected from at least 10 independent devices.

Table S2. The PCE statistical data based on different D:A ratio.

D:A ratio	V_{OC} (V)	J_{SC} (mA/cm^2)	FF (%)	PCE (%)
1:1.2	0.878	22.35	75.05	14.74
	0.873±0.003	22.41±0.33	73.26±1.18	14.33±0.18
1:1.5	0.880	22.36	76.95	15.14
	0.873±0.005	22.63±0.33	74.81±0.83	14.78±0.16
1:2	0.873	22.03	75.68	14.55
	0.872±0.004	22.21±0.34	74.08±1.29	14.34±0.15

The data were collected from at least 10 independent devices.

Table S3. The PCE statistical data based on two different additives.

Additives	V_{OC} (V)	J_{SC} (mA/cm^2)	FF (%)	PCE (%)
CN	0.880	22.36	76.95	15.14
	0.873±0.005	22.63±0.33	74.81±0.83	14.78±0.16
DIB	0.879	22.95	77.39	15.61
	0.878±0.005	22.55±0.80	75.34±2.10	14.91±0.32

The data were collected from at least 10 independent devices.

Table S4. The 42 cm^2 module PCE statistical data based on different modification layers.

Electrode	V_{OC} (V)	J_{SC} (mA/cm^2)	FF (%)	PCE (%)
AgCu/LITO	6.247	3.28	76.18	15.60
	6.211±0.038	3.23±0.03	75.42±0.59	15.13±0.24
Ag/PH1000	6.145	3.30	69.57	14.11
	6.060±0.056	3.20±0.06	69.33±0.55	13.45±0.40

The data were collected from at least 5 independent modules.

Table S5. The PCE statistical data based on different modification layers.

Electrode	V_{OC} (V)	J_{SC} (mA/cm^2)	FF (%)	PCE (%)
AgCu/LITO	0.895	24.38	78.42	17.12
	0.888±0.003	23.84±0.40	78.01±0.64	16.52±0.27
Ag/PH1000	0.879	22.95	77.39	15.61
	0.878±0.005	22.55±0.80	75.34±2.10	14.91±0.32

The data were collected from at least 10 independent devices.

Table S6. The sheet resistances of LITO modification layers with different In:Sn ratios and thicknesses on PET substrate.

Modification Layer		Sheet Resistances (Ω/□)
In:Sn	Thickness (nm)	
LITO (90:10)	30	155.9
	20	253.7
	30	160.1
LITO (93:7)	60	87.6
	90	82.4
	135	76.7

Table S7. The sheet resistances of two different modification layers on PET substrate.

Modification Layer	Sheet Resistances (Ω/□)				
	No.1	No.2	No.3	No.4	No.5
PH1000	2330	2490	2090	2090	1880
LITO (93:7)	157.4	157.8	159.7	160.0	160.5

Table S8. The PCE statistical data based on two different electrodes.

Modification Layer	V_{OC} (V)	J_{SC} (mA/cm^2)	FF (%)	PCE (%)
PH1000	0.886	20.31	65.73	11.83
	0.885±0.002	20.57±0.39	62.28±2.18	11.33±0.28
LITO (90:10)-	0.887	23.00	77.81	15.87
30 nm	0.888±0.002	22.67±0.26	76.48±2.72	15.40±0.62
LITO (93:7)-	0.879	24.35	76.76	16.44
20 nm	0.878±0.005	24.40±0.37	75.71±0.87	16.22±0.12
LITO (93:7)-	0.895	24.38	78.42	17.12
30 nm	0.888±0.003	23.84±0.40	78.01±0.64	16.52±0.27

The data were collected from at least 10 independent devices.

Table S9. The comparison of AgCu/LITO with reported state-of-the-art TCEs.

Electrode	Opto-electrical property	*FoM*	Reference
AgNWs	15 Ω/□, 83%[a]	129	*Nat. Photon.* **7**, 817–824 (2013).
	12 Ω/□, 84%[a]	172	*J. Am. Chem. Soc.* **144**, 8658–8668 (2022).
	43 Ω/□, 85%[a]	52	*Adv. Funct. Mater.* **34**, 2408453 (2024).
Ag-mesh/PH1000	3 Ω/□, 84%[a]	690	*Nat. Commun.* **7**, 10214 (2016)
PEDOT:PSS	75 Ω/□, 86%[a]	32	*Adv. Mater.* **30**, 1800075 (2018).
Graphene	54 Ω/□, 91%[b]	72	*Nat. Photon.* **6**, 105–110 (2012).
Cu nanotrough network	2 Ω/□, 90%[b]	1742	*Nat. Nanotech.* **8**, 421–425 (2013).
FlexAgNE	10Ω/□, 92%[b]	416	*Nat. Electron.* **2**, 513–520 (2019).
**AgCu/LITO**	**0.85 Ω/□, 90%[b]**	**4188**	**This work**

[a] The transmittance data reported here include contributions from the substrate (i.e., measured with air as reference).

[b] The transmittance data reported here exclude contributions from the substrate (i.e., measured with the substrate as reference).

Table S10. The comparison of AgCu/LITO with reported state-of-the-art TCEs.

Modification Layer	Bending cycles 0	100	200	300	400	500	600	700	800	900	1000
LITO (90:10)-30 nm	1.17	1.31	1.36	1.43	1.39	1.31	1.5	2.24	1.93	1.43	2.29
LITO (93:7)-20 nm	0.87	0.75	0.72	0.71	0.72	0.97	0.97	0.83	0.67	0.84	0.78
LITO (93:7)-30 nm	0.90	1.05	1.27	1.30	1.51	1.42	1.40	1.47	1.28	1.04	1.15
LITO (93:7)-60 nm	0.69	0.97	0.89	0.72	0.95	1.18	1.95	2.24	3.79	67	65.98
LITO (93:7)-90 nm	0.8	0.74	2.34	3.41	2.47	61.27	72.88	554	873	686	1422
LITO (93:7)-135 nm	0.75	0.54	0.65	26.59	1039	2084	1314	2018	2228	3891	6661

Table S11. Power supply and consumption statistics of each component of SUAVs.

Item	Type	Power (W)
Solar Panel	Supply	3.66
Li-ion Battery	Supply	3.74
GPS Module	Consumption	0.07
Micro Controller	Consumption	0.17
Environment Sensor	Consumption	0.06
Propeller	Consumption	5.55
Steering engine	Consumption	1.11
Receiver	Consumption	0.37
Gyroscope	Consumption	0.07

References

1. Chen X, Xu G, Zeng G *et al.* Realizing ultrahigh mechanical flexibility and >15% efficiency of flexible organic solar cells via a "welding" flexible transparent electrode. *Adv Mater* 2020; **32**: 1908478.
2. Liu X, Zheng Z, Wang J *et al.* Fluidic manipulating of printable zinc oxide for flexible organic solar cells. *Adv Mater* 2022; **34**: 2106453.
3. Han Y, Hu Z, Zha W *et al.* 12.42% Monolithic 25.42 cm^2 flexible organic solar cells enabled by an amorphous ITO-modified metal grid electrode. *Adv Mater* 2022; **34**: 2110276.
4. Qin F, Sun L, Chen H *et al.* 54 cm^2 large-area flexible organic solar modules with efficiency above 13%. *Adv Mater* 2021; **33**: 2103017.
5. Wang G, Zhang J, Yang C *et al.* Synergistic optimization enables large-area flexible organic solar cells to maintain over 98% PCE of the small-area rigid devices. *Adv Mater* 2020; **32**: 2005153.
6. Zhang J, Mao H, Zhou K *et al.* Polymer-entangled spontaneous pseudo-planar heterojunction for constructing efficient flexible organic solar cells. *Adv Mater* 2024; **36**: 2309379.
7. Zheng X, Zuo L, Zhao F *et al.* High-efficiency ITO-free organic photovoltaics with superior flexibility and upscalability. *Adv Mater* 2022; **34**: 2200044.
8. Han Y, Chen X, Wei J *et al.* Efficiency above 12% for 1 cm^2 flexible organic solar cells with Ag/Cu grid transparent conducting electrode. *Adv Sci* 2019; **6**: 1901490.
9. Liu Y, Qin F, Wang Y *et al.* Large‐area flexible organic photovoltaic modules on smoothened silver nanowire transparent electrodes with thick electron transporting layer. *Adv Funct Mater* 2024; **34**: 2408453.
10. Wang Z, Han Y, Yan L *et al.* High power conversion efficiency of 13.61% for 1 cm^2 flexible polymer solar cells based on patternable and mass-producible gravure-printed silver nanowire electrodes. *Adv Funct Mater* 2020; **31**: 2007276.
11. Xie C, Liu Y, Wei W *et al.* Large‐area flexible organic solar cells with a robust silver nanowire-polymer composite as transparent top electrode. *Adv Funct Mater* 2022; **33**: 2210675.
12. Shen YF, Zhang H, Zhang J *et al.* In situ absorption characterization guided slot-die-coated high-performance large-area flexible organic solar cells and modules. *Adv Mater* 2023; **35**: 2209030.
13. Tian C, Zhang J, Shen Y *et al.* Mitigating coffee ring effects for efficient upscaling of flexible organic solar cells. *Solar RRL* 2023; **7**: 2300349.
14. Shan T, Wang Y, Chen Q *et al.* Achieving efficient flexible and large-area organic solar cells via additive-assisted fluorous solvent soaking. *Chem Eng J* 2023; **475**: 146038.
15. Wang Z, Guo J, Pan Y *et al.* Manipulating the macroscopic and microscopic morphology of large‐area

gravure-printed ZnO films for high-performance flexible organic solar cells. *Energy Environ Mater* 2023; **7**: e12592.

16. Zeng G, Chen W, Chen X *et al.* Realizing 17.5% efficiency flexible organic solar cells via atomic-level chemical welding of silver nanowire electrodes. *J Am Chem Soc* 2022; **144**: 8658-68.

17. Pan W, Han Y, Wang Z *et al.* An efficiency of 14.29% and 13.08% for 1 cm^2 and 4 cm^2 flexible organic solar cells enabled by sol–gel ZnO and ZnO nanoparticle bilayer electron transporting layers. *J Mater Chem A* 2021; **9**: 16889-97.

18. Mao L, Chen Q, Li Y *et al.* Flexible silver grid/PEDOT:PSS hybrid electrodes for large area inverted polymer solar cells. *Nano Energy*. 2014; **10**: 259-67.

19. Xie C, Xiao C, Fang J *et al.* Core/shell AgNWs@SnOx electrodes for high performance flexible indoor organic solar cells with >25% efficiency. *Nano Energy*. 2023; **107**: 108153.

20. Lu X, Xie C, Liu Y *et al.* Increase in the efficiency and stability of large-area flexible organic photovoltaic modules via improved electrical contact. *Nat Energy* 2024; **9**: 793-802.

21. Zhang J, Zhao Y, Fang J *et al.* Enhancing performance of large-area organic solar cells with thick film via ternary strategy. *Small* 2017; **13**: 1700388.

22. Wang C, Ma X, Shen Y-f *et al.* Unique assembly of giant star-shaped trimer enables non-halogen solvent-fabricated, thermal stable, and efficient organic solar cells. *Joule* 2023; **7**: 2386-401.
